# Plasma ceramide Cer24:0 and insulin resistance: associations with TyG and TG/HDL-C in a multicenter study of coronary artery disease cohorts

**DOI:** 10.3389/fendo.2026.1777380

**Published:** 2026-02-26

**Authors:** Shuanli Xin, Rui Wang, Xuejiao Chen, Chao Chang, Xiufeng Zhao, Yong Zeng, Liang Zhang, Mengdan Miao, Guidong Wang, Xiaopeng Li, Junwei Wang, Xin Zhang, Zhijiang Xie

**Affiliations:** 1 Department of Cardiology, Handan First Hospital, Handan, Hebei, China; 2 Department of Cardiology, The First Affiliated Hospital of Hebei North University, Zhangjiakou, Hebei, China; 3 Department of Cardiology, Affiliated Hospital of Hebei Engineering University, Handan, Hebei, China; 4 Department of Cardiology, Beijing Anzhen Hospital, Beijing, China

**Keywords:** causal forest DML, ceramides, coronary artery disease, insulin resistance, mixed graphical models

## Abstract

**Background:**

Insulin resistance (IR) is a key metabolic determinant of cardiovascular risk. Ceramides, a class of bioactive lipids, have been linked to IR; however, their clinical associations in patients with established coronary artery disease (CAD) are incompletely characterized.

**Methods:**

In a prospective, multicenter observational cohort of adults with established CAD (n=987), we quantified plasma ceramide species (Cer16:0, Cer18:0, Cer24:0, Cer24:1) and surrogate IR indices (triglyceride–glucose index (TyG), metabolic score for insulin resistance (METS-IR), and triglyceride–to–high-density lipoprotein cholesterol ratio (TG/HDL-C)). Mixed graphical models (MGM) were used to estimate conditional associations within a multivariable network. Double machine learning (DML) with causal-forest estimators provided covariate-adjusted association estimates and probed robustness values (RV) to unmeasured confounding. Exposures were standardized per standard deviation. We also developed a ceramide-augmented model to classify clinical IR, prespecified as TyG ≥ 9, and quantified discrimination by the area under the receiver operating characteristic curve (ROC–AUC).

**Results:**

Ceramides correlated with IR indices. In MGM analyses, Cer24:0 showed a direct conditional association with TyG (partial r=0.23; 95% CI, 0.17–0.29), independent of other variables in the network. In DML analyses, per 1-unit increase in ln (Cer24:0) was associated with higher TyG (estimate, 0.459; 95% CI, 0.252–0.665; P = 0.001); These estimates demonstrated moderate RV to unmeasured confounding, with an RV (*theta*) of 0.223. A ceramide-augmented model classified clinical IR with an ROC–AUC of 0.770 (95% CI, 0.741–0.799).

**Conclusions:**

Across complementary analytic frameworks, Cer24:0 consistently exhibited positive associations with lipid-centric IR metrics among adults with established CAD. Although observational, these findings suggest that circulating ceramide profiling—particularly Cer24:0—may refine metabolic risk stratification beyond conventional indices in cardiology practice.

## 1 Introduction

Coronary artery disease (CAD) continues to be among the foremost causes of mortality worldwide (1). While traditional risk factors such as age, sex, hypertension, and altered glucose metabolism remain important, recent research has increasingly underscored the role of metabolic dysregulation, particularly insulin resistance (IR), as a fundamental driving force in the progression of atherosclerosis and the occurrence of adverse cardiovascular events (2, 3). Bioactive lipid networks, especially ceramides, are believed to play a critical intermediary role in the relationship between IR and CAD, potentially serving as proximal biomarkers and participating in key pathological processes (4, 5).

Despite mounting evidence connecting ceramides to IR and CAD, the biological effects of ceramides demonstrate stage-dependency and contextual-variability (6, 7). This suggests that ceramides may play different roles at various stages of disease progression, underscoring the necessity for longitudinal studies to elucidate their dynamic interactions with proximal signals and downstream effects.

In the assessment of IR, the hyperinsulinemia-euglycemic clamp is recognized as the gold standard; however, its substantial cost and operational complexity limit its routine clinical use (8). As a result, alternative indices derived from standard laboratory tests, such as the triglyceride-glucose index (TyG), the metabolic score for insulin resistance (METS-IR), and the triglyceride/high-density lipoprotein cholesterol ratio (TG/HDL-C), have been developed as practical tools for predicting metabolic risk and cardiovascular events (9, 10).

However, the role of ceramides in metabolism is characterized by bidirectionality and context dependency (11). On one hand, Cer24:0 is often positively correlated with IR metrics, suggesting it may act as a signaling molecule for metabolic abnormalities (12, 13); conversely, within CAD patients, elevated Cer24:0 levels are associated with more favorable prognoses, indicating that the same molecule may engage in divergent biological pathways during the downstream outcome phase and even possess potential protective signaling (14). This paradox underscores the bidirectional information carried by ceramide lipidome in risk stratification, necessitating longitudinal studies and multi-outcome analyses for clarification. Given this, the current study aims to investigate the associations between ceramide profiles and surrogate IR indices and to evaluate their potential value in identifying IR among patients with CAD, laying the groundwork for future research into their relationship with clinical outcomes.

## 2 Methods

### 2.1 Study design and participants

This investigation was structured as a prospective, multicenter, observational study conducted at Beijing Anzhen Hospital and Handan First Hospital from April 1, 2021, to August 31, 2022. The study protocol was registered with the Chinese Clinical Trial Registry (ChiCTR-2200056697) prior to the enrollment of the initial participant. Ethical approval was secured from the institutional review boards of all participating centers. Written informed consent was obtained from each participant before the initiation of any study-specific procedures.

The study population consisted of adults aged 18 years and older with suspected CAD who were scheduled for coronary imaging, irrespective of sex or ethnicity. Key exclusion criteria included: (1) non-coronary cardiac disorders (e.g., valvular or congenital disease), (2) suspected aortic coarctation or acute pulmonary embolism, (3) familial hypercholesterolemia, (4) substance misuse or alcohol dependence, (5) cerebrovascular accident or major cardiac intervention within the preceding 3 months, and (6) any condition deemed by the treating physician to preclude safe participation.

### 2.2 Assessment of IR

IR was quantified by three validated surrogate indices


$$TyG=\text{ln}\left(\frac{\text{TG}\left(\text{mg}/\text{dL}\right)\times \text{FBG}\left(\text{mg}/\text{dL}\right)}{2}\right)$$

$$METS-IR=\ln\left[2\times FBG\;(mg/dL)+TG\;(mg/dL)\right]\times \frac{BMI}{\ln\left(HDL-C\;(mg/dL)\right)}$$

$$TG\left(mg/dL\right)/HDL-C\left(mg/dL\right)=\frac{TG\left(mg/dL\right)}{HDL-C\left(mg/dL\right)}$$

### 2.3 Ceramide quantification and selection rationale

Venous blood samples were collected following an overnight fast and placed into EDTA tubes, which were subsequently stored at 4 °C for a maximum duration of 8 hours prior to centrifugation at 1000–1200 g for 15 minutes at 4 °C. The resulting plasma aliquots were then frozen at –80 °C for a period not exceeding 14 days. Quantification of four ceramide species—Cer16:0, Cer18:0, Cer24:0, and Cer24:1—was performed using liquid chromatography–tandem mass spectrometry (LC–MS/MS; AB Sciex API 4500 MD, SCIEX, USA) in accordance with established protocols. Quality control criteria were defined as a relative deviation of ≤ ± 15%, with a permissible deviation of ≤ ± 20% for low concentration controls.

We quantified four specific ceramide species—Cer16:0, Cer18:0, Cer24:0, and Cer24:1—based on several considerations. Firstly, these species represent the predominant fraction of plasma ceramides, collectively constituting over 80% of the total circulating ceramides, thereby offering a comprehensive representation of the principal ceramide pool pertinent to cardiometabolic diseases (15). Secondly, these four species have distinct primary biosynthetic origins: Cer16:0 is predominantly synthesized by ceramide synthase 6 (CerS6), Cer18:0 by CerS1, and Cer24:0 by CerS2. This enzymatic specificity implies that these ceramides may reflect fundamentally distinct metabolic pathways and biological functions (16, 17). Extensive evidence shows that Cer16:0 and Cer18:0 are linked to higher cardiovascular risk and insulin resistance, while Cer24:0 may have neutral or protective effects. This makes the four-ceramide panel valuable for studying metabolic issues in CAD patients. Additionally, the panel aligns with commercial cardiovascular risk tools and has been validated in large studies like the Framingham Heart Study and EPIC-Potsdam (18, 19).

### 2.4 Association analyses

Owing to the non-normal distribution of ceramide concentrations, pairwise associations with IR indices were evaluated using two-tailed Spearman rank correlation. The study explored the conditional independence among IR indices and ceramide species through the application of a mixed graphical model (MGM). An *ℓ*_1_-penalized neighborhood regression was employed for each node, with the global tuning parameter determined via the extended Bayesian information criterion (EBIC, *γ = 0.1*), which resulted in a sparse network. In the resulting undirected graph, each variable is depicted as a node, and an edge denotes a non-zero partial correlation, reflecting a direct dependency after controlling for all other variables. Statistical uncertainty was assessed using 1,000 non-parametric bootstrap resamples, and 95% confidence intervals (CIs) were computed for each edge weight.

Potential non-linear dose–response relationships between ceramide concentrations and indices of IR were examined using restricted cubic spline regression (RCS) with four knots strategically placed at the 5th, 35th, 65th, and 95th percentiles. The multivariable models were adjusted for pertinent covariates. Global associations and non-linear relationships were assessed using Wald χ² tests.

### 2.5 Causal effect estimation and sensitivity analysis via double machine learning

We used double machine learning (DML) for a continuous treatment to estimate the average partial effect (APE) of the natural log–transformed ceramide concentration on IR, adjusting for measured confounders. The outcome Y was a continuous measure of IR. The treatment T was the natural logarithm of ceramide concentration. covariates listed in **Table 1** were included as potential confounders.

**Table 1:** Baseline clinical characteristics.

Characteristics	N=987
Age (years)	65.1 (57.4–71.9)
BMI (kg/m^2^)	24.6 (21.9–27.4)
Male	681 (69.0%)
DM	302 (30.6%)
CVD	83 (8.4%)
HTN	602 (61.0%)
CKMB (ng/mL)	1.6 (1.1–2.5)
BNP (ng/L)	41.0 (20.0–89.0)
AST (U/L)	20.0 (16.0–26.0)
ALT (U/L)	20.0 (14.0–30.0)
TBIL (μmol/L)	11.0 (8.6–14.6)
Cr (μmol/L)	70.1 (61.2–80.1)
FBG (mg/dL)	106.6 (92.3–139.4)
TC (mg/dL)	153.9 (131.3–182.7)
TG (mg/dL)	127.5 (93.9–183.3)
LDL-C(mg/dL)	88.2 (69.0–111.9)
HDL-C(mg/dL)	40.6 (34.8–48.3)
hsCRP (mg/L)	1.2 (0.6–2.9)
Cer16:0(ng/mL)	165.0 (126.5–212.0)
Cer18:0(ng/mL)	44.8 (32.1–61.6)
Cer24:1(ng/mL)	497.0 (373.0–663.0)
Cer24:0(ng/mL)	1800.0 (1400.0–2270.0)
TyG	8.9 (8.5–9.3)
TG/HDL-C	3.2 (2.1–4.8)
METS-IR	39.3 (34.0–45.9)

ALT, Alanine Aminotransferase; AST, Aspartate Aminotransferase; BMI, Body Mass Index; BNP, Brain Natriuretic Peptide; Cer, Ceramide; CKMB, Creatine Kinase-MB; Cr, Creatinine; CVD, Cerebrovascular Disease; DM, Diabetes Mellitus; FBG, Fasting Blood Glucose; HDL-C, High-Density Lipoprotein Cholesterol; hsCRP, high-sensitivity C-Reactive Protein; HTN, Hypertension; LDL-C, Low-Density Lipoprotein Cholesterol; METS-IR, Metabolic Score for Insulin Resistance; TBIL, Total Bilirubin; TC, Total Cholesterol; TG, Triglycerides; TG/HDL-C, Triglyceride-HDL-C Ratio. TyG, Triglyceride-Glucose Index.

Nuisance functions for the outcome E[Y|X] and the treatment E[T|X] were modeled using a random forest regressor (scikit-learn RandomForestRegressor). We implemented cross-fitting and orthogonalization and trained a causal forest for continuous treatments with 1,000 trees and prespecified honesty (sample splitting), subsampling, and minimum leaf-size constraints. Hyperparameters were prespecified; in sensitivity analyses, they were selected by cross-validation. After model fitting, we estimated the adjusted APE per 1−unit increase in T= ln (Ceramide) on Y and its Wald−type 95% CIs using the estimator’s asymptotic variance.

To evaluate the potential impact of unmeasured confounding on the primary estimand, we performed a prespecified sensitivity analysis based on Cinelli’s framework that indexes confounding strength using partial R-squared for the residualized treatment and outcome (20). Under an equal−strength assumption that places the same maximum allowable confounding on both sides, we report the robustness value defined as the smallest confounding strength on this common scale that would cause the 95% CIs to include the null. We also present confounding−adjusted intervals across a prespecified grid of confounding strengths under the equal−strength assumption. In this study, *theta* represents the adjusted average treatment effect of ceramide species on parameters related to insulin resistance, after accounting for hypothesized unmeasured confounders. Additionally, two robustness metrics are provided to assess sensitivity to hidden bias: (1) RV(*theta*), which reflects the minimum strength of association that an unmeasured confounder must simultaneously possess with both the treatment and outcome variables to drive the point estimate *theta* to the null; and (2) RV(CI), which specifies the minimum confounding strength necessary to shift the confidence interval boundary to include the null value, thereby negating statistical significance.

### 2.6 Machine-learning modelling

In this study, six machine learning algorithms were implemented utilizing H2O version 3.42.0.4: Generalized Linear Model (GLM), Gradient Boosting Machine (GBM), Deep Learning (DL), Extreme Gradient Boosting (XGBoost), Distributed Random Forest (DRF), and Extremely Randomized Trees (XRT). Hyperparameter optimization was performed through a grid search strategy, integrated with five-fold cross-validation on the derivation dataset. For classification tasks, the primary evaluation metric was the area under the receiver operating characteristic curve (ROC–AUC), while secondary metrics included the area under the precision recall curve (PR–AUC), mean per-class error, and log loss. In regression tasks, the primary evaluation metrics were Mean Absolute Error (MAE), Root Mean Square Logarithmic Error (RMSLE), and mean residual deviance. Metrics applicable to both classification and regression tasks comprised Root Mean Square Error (RMSE) and Mean Squared Error (MSE). To mitigate class imbalance in classification tasks, class-balanced loss functions were utilized.

The dataset was systematically divided into five equal subsets, referred to as folds. During each iteration, one-fold was designated as the validation set, while the remaining four folds were combined to form the training set. The model was trained on these four training folds and subsequently evaluated on the reserved validation fold to assess its performance. For each validation set across all five folds, we calculated several key performance metrics, including accuracy, ROC–AUC, precision, recall, F1 score, RMSE, and specificity.

To assess the overall feature importance within machine learning models demonstrating optimal predictive performance, SHAP (SHapley Additive exPlanations) values were employed. This innovative approach utilizes game theory to integrate individual feature contributions, thereby enhancing the interpretation of the model’s global behavior. We used SHAP to quantify both global and local feature importance. A positive SHAP value indicates an increased likelihood of predicting IR.

### 2.7 Statistical analyses

Baseline data were reported as mean ± standard deviation for continuous variables that followed a normal distribution, and these were analyzed using an independent samples t-test. For continuous variables that did not conform to a normal distribution, data were presented as median with interquartile range and analyzed using the Mann-Whitney U-test. Categorical variables were expressed as frequencies and analyzed using either the Chi-square test or Fisher’s exact test for group comparisons.

The study conducted a thorough evaluation of the model’s discriminative ability and calibration, utilizing the ROC–AUC and PR–AUC metrics. To assess the statistical and clinical enhancement in predictive capability afforded by the integration of ceramide species into the baseline model, we utilized the Net Reclassification Improvement (NRI) metric. Calibration was assessed through calibration plots and by calculating the calibration intercept and slope. A two-tailed P-value of less than 0.05 was considered indicative of statistical significance, with 95% CIs derived through 1000-fold bootstrap resampling. All analyses were performed using R and Python.

## 3 Results

### 3.1 Subject characteristics

The baseline characteristics of the study population are comprehensively outlined in **Table 1**. The cohort consisted of 987 participants with a median age of 65.1 years, of whom 69% were male. The participants were slightly overweight, with a median body mass index (BMI) of 24.6 kg/m² and exhibited a notable prevalence of cardiometabolic conditions: 61% had hypertension, 30.6% had diabetes mellitus, 8.4% had established cerebrovascular disease. Routine biochemical assessments indicated preserved renal and hepatic function, modest systemic inflammation, and lipid profiles that were, on average, borderline dyslipidemia. Indices of IR, including fasting glucose levels, the TyG, the TG/HDL-C, and the METS-IR, were mildly elevated. Median levels of B-type natriuretic peptide and creatine kinase-MB were within clinically accepted reference ranges, reflecting the ambulatory nature of the study population. Targeted lipidomic analysis revealed a wide distribution of saturated and very long chain ceramides (VLC), with median concentrations of the four specified species (Cer16:0, Cer18:0, Cer24:1, and Cer24:0) detailed in **Table 1**.

### 3.2 Association analyses

#### 3.2.1 Spearman correlation analysis

The Spearman rank-order analysis identified significant and predominantly positive correlations between circulating ceramide species and surrogate markers of IR (**Supplementary Figure 1**). The inter-ceramide correlations were robust, with correlation coefficients ranging from 0.39 to 0.79 (P< 0.05). Among the individual ceramide species, Cer24:0 demonstrated the strongest association with the TyG (r = 0.37; P< 0.05). Additionally, both Cer18:0 and Cer24:1 were correlated with the TyG (r = 0.35 and 0.34, respectively) as well as the TG/HDL-C (r = 0.35 and 0.33, respectively; P< 0.05 for all). In contrast, Cer16:0 exhibited weaker associations with markers of IR and was not significantly associated with METS-IR (r = 0.06; P = 0.053).

#### 3.2.2 MGM analysis

**Figure 1:**
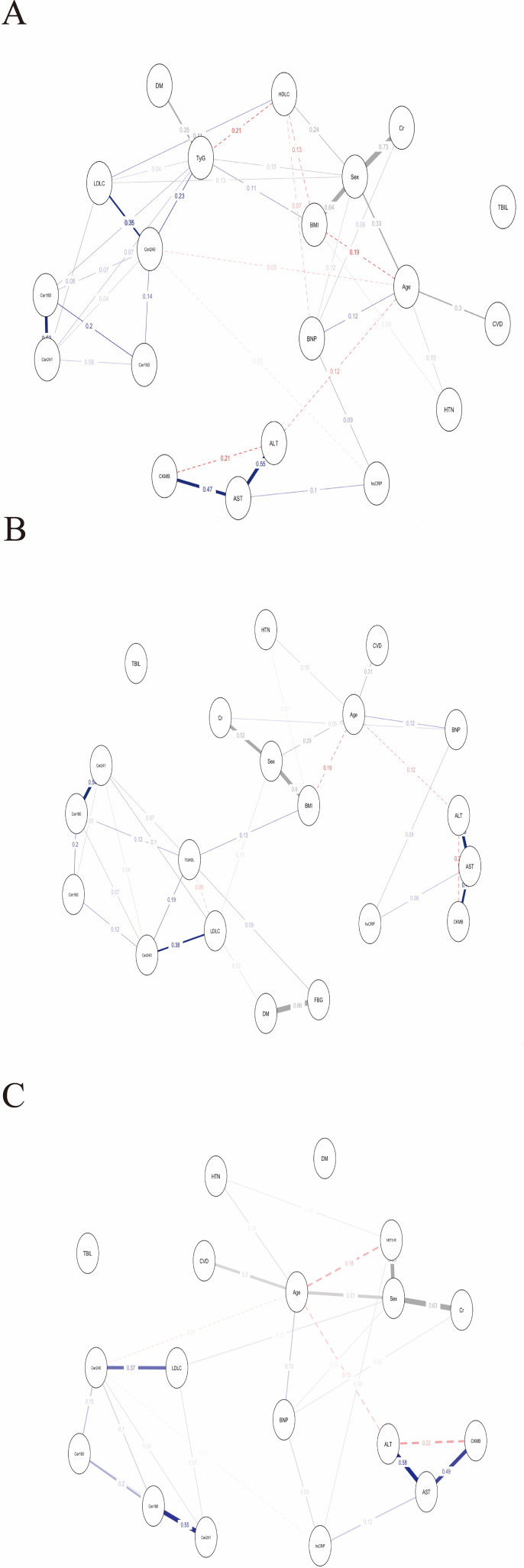
Mixed graphical model networks of conditional dependencies between plasma ceramides and three indices of insulin resistance. **(A)** TyG; **(B)** TG/HDL−C; **(C)** METS−IR. The networks were estimated using a mixed graphical model including both continuous and categorical variables. Edges, which are the lines connecting nodes, signify direct, non-zero conditional dependencies between two variables. This indicates the presence of a statistically significant relationship between these variables, even after accounting for the influence of all other variables within the network, a process known as sparse estimation. The thickness of an edge represents the magnitude of the association, while the color of an edge conveys the nature of the relationship: solid blue edges denote a positive correlation, dashed red edges signify a negative correlation, and grey edges represent associations involving categorical variables.

Using MGM, we constructed a conditional-dependency network encompassing four plasma ceramide species, three surrogate indices of IR, and conventional metabolic variables (**Figure 1**). Partial correlation coefficients were utilized to quantify direct associations between nodes while controlling for all other variables.

The four ceramide species were interconnected, with their conditional dependencies exhibiting a distinct hierarchical structure. The central and most robust connection was identified between Cer18:0 and Cer24:1, with partial correlation coefficients ranging from 0.53 to 0.55 across all models; this pair constituted the structural backbone of the network after accounting for the influence of the other ceramides. Secondary connections were relatively modest. In representative models, Cer16:0 and Cer24:0 demonstrated statistically significant, albeit weaker, conditional correlations with additional network components, with partial correlation coefficients ranging from 0.07 to 0.20.

In analyses adjusted for multiple variables and employing partial-correlation techniques, the associations between ceramide species and surrogate indicators of IR were found to vary according to chain length and saturation. Specifically, Cer24:0 demonstrated a positive association with the TyG (partial r = 0.23; 95% CI, 0.17 to 0.29). In contrast, the associations for Cer18:0 (partial r = 0.08; 95% CI, 0.00 to 0.13) and Cer24:1 (partial r = 0.07; 95% CI, 0.00 to 0.11) were comparatively weaker, and no association was observed for Cer16:0. These results were corroborated by forest-plot summaries. Similar patterns were evident with respect to the TG/HDL-C, where Cer24:0 exhibited a moderate positive association (partial r = 0.19; 95% CI, 0.11 to 0.27), while the associations for Cer18:0 (partial r = 0.12; 95% CI, 0.00 to 0.18) and Cer24:1 (partial r = 0.00; 95% CI, 0.00 to 0.14) were less pronounced. Furthermore, network analyses revealed no direct conditional dependencies between ceramide subspecies and METS-IR. Forest plots displaying point estimates and 95% CIs of MGM edge weights (partial correlations) involving TyG, TG/HDL−C, and METS−IR are provided in the **Supplementary Figure 2–4**.

### 3.3 RCS regression analysis

**Figure 2:**
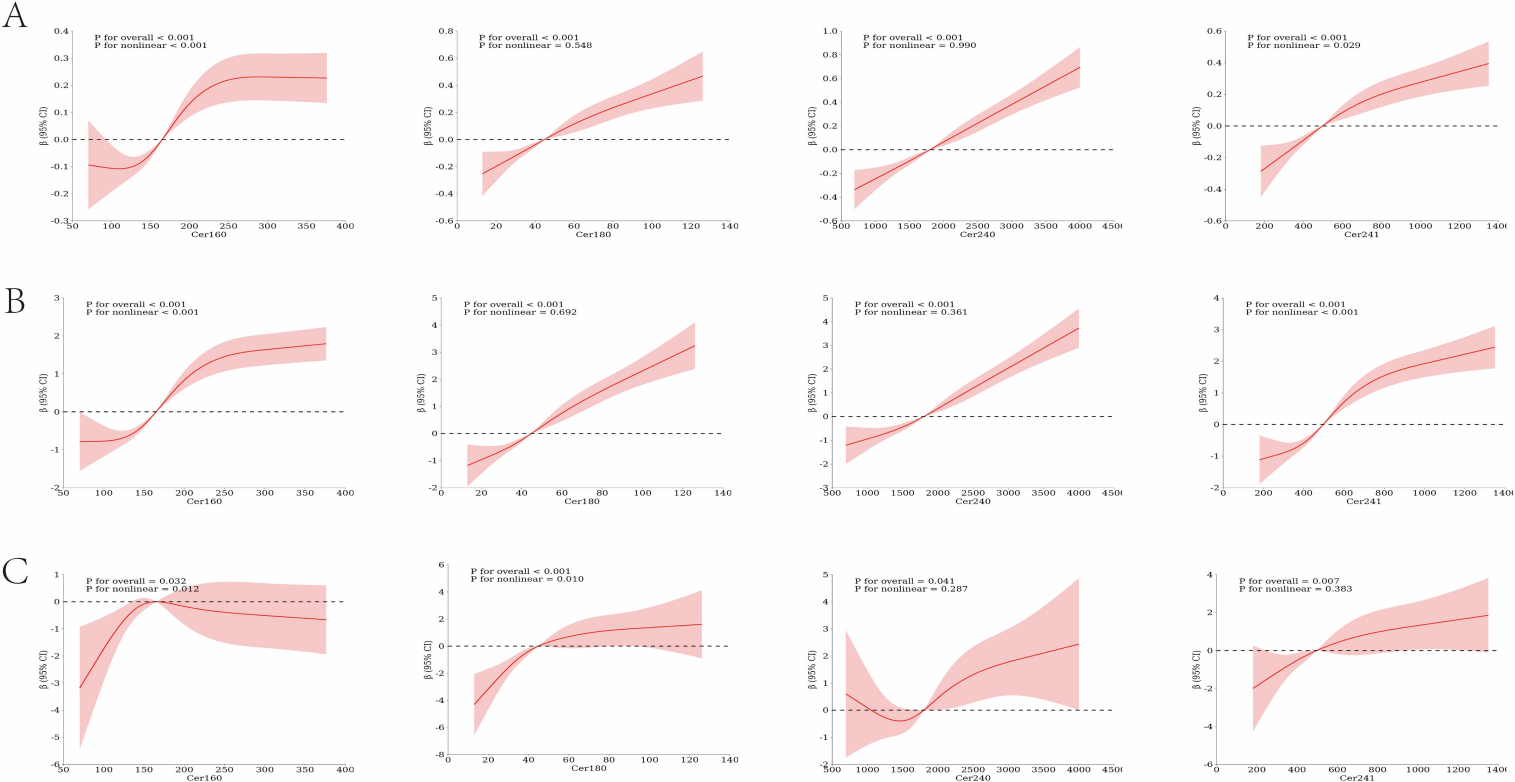
Restricted cubic spline (RCS) analyses showing multivariable-adjusted associations between plasma ceramide concentrations and indices of insulin resistance. **(A)** TyG; **(B)** TG/HDL-C; **(C)** METS-IR.

RCS regression analysis demonstrated concentration-dependent associations between circulating ceramide species and various indices of IR. In the unadjusted analyses, all ceramide species under investigation showed significant correlations with the TyG (overall P< 0.001), exhibiting nonlinear dose-response patterns for all species except Cer24:0 (P for nonlinearity = 0.88) (**Supplementary Figure 5**). After adjusting for age, sex, body mass index, and additional predetermined covariates, Cer16:0, Cer18:0, Cer24:0, and Cer24:1 continued to exhibit significant associations with the TyG (overall P< 0.01). Nonlinear relationships persisted for Cer16:0 (P< 0.001 for nonlinearity) and Cer24:1 (P = 0.029 for nonlinearity). Similar concentration-dependent associations were also identified between ceramide species and the TG/HDL-C, as well as the METS-IR (**Figure 2**).

### 3.4 DML causal effect estimates with sensitivity analyses

Using an orthogonal machine learning framework with covariate adjustment, we estimated average partial effects of metabolic exposures on IR. As shown in **Table 2**, TyG exhibited a statistically significant positive adjusted association per 1-unit increase in ln (Cer24:0) (estimate 0.459; 95% CI, 0.252 to 0.665; P = 0.001). TG/HDL-C also showed a significant positive adjusted association per 1-unit increase (estimate, 0.627; 95% CI, 0.460 to 0.885; P = 0.001). In contrast, the adjusted association of METS−IR was not statistically significant (estimate, 1.635; 95% CI, −1.366 to 4.636; P = 0.286). Sensitivity analyses for unmeasured confounding indicated moderate robustness values (RV) for TyG and TG/HDL-C but minimal RV for METS−IR; RV are reported in **Table 2**.

**Table 2:** Average partial effects of ceramide species on insulin resistance indices and sensitivity analysis results.

Index	Original analysis					Sensitivity analysis			
	Mean point	SE	zstat	p value	95%CI	Theta	95%CI	RV (Theta)	RV (CI)
TyG	0.459	0.105	3.395	0.001	0.252–0.665	0.461	0.245-0.675	0.223	0.168
TG/HDL-C	0.627	0.108	3.313	0.001	0.460–0.885	0.671	0.477–0.866	0.330	0.283
METS-IR	1.635	1.531	0.469	0.286	-1.366–4.636	1.658	-1.523–4.810	0.059	0

CI, confidence interval; METS-IR, metabolic score for insulin resistance; RV, robustness value; SE, standard error; TG/HDL-C, triglyceride-to-HDL cholesterol ratio; TyG, triglyceride-glucose index.

### 3.5 SHAP analysis

**Figure 3:**
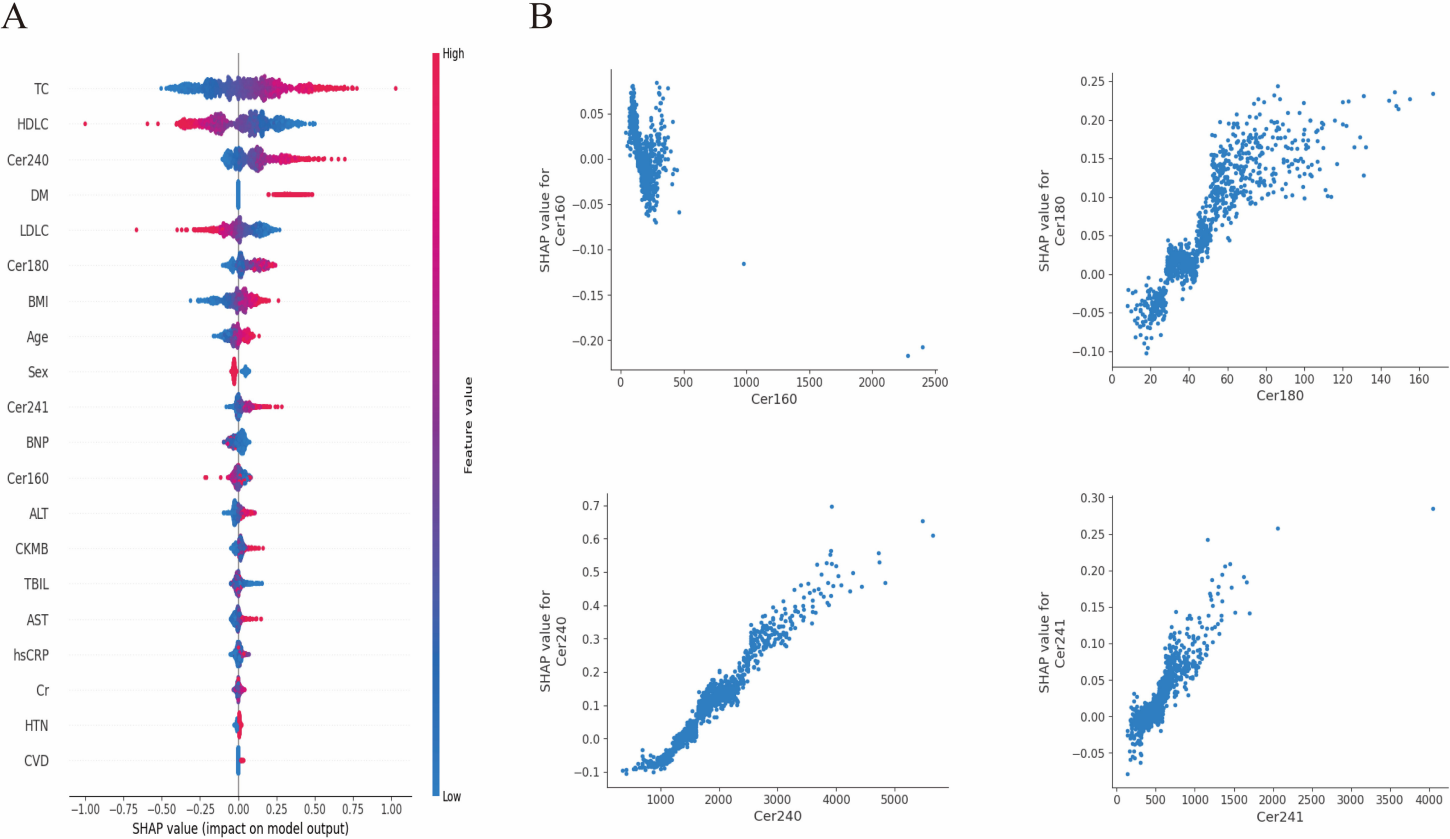
SHAP Analysis for Prediction of the TyG. **(A)** SHAP Summary Plot: The features are ordered according to their mean absolute SHAP values, indicating their relative importance. The x-axis represents the SHAP values, which reflect the impact on the predicted TyG. Positive SHAP values (to the right of zero) suggest an increase in the TyG, whereas negative values (to the left of zero) suggest a decrease. The color of the points indicates the actual value of the feature, with red representing high values and blue representing low values. **(B)** SHAP Dependence Plots: These plots demonstrate the relationship between specific feature values (x-axis) and their corresponding SHAP values (y-axis). For Cer16:0 (Top-left), lower values tend to have a slight negative impact on the predicted TyG. For Cer18:0 (Top-right), increasing levels are positively correlated with SHAP values, thereby contributing to a higher predicted TyG. Cer24:0 (Bottom-left) shows that elevated levels have a strong positive correlation with SHAP values, significantly enhancing the predicted TyG. Finally, for Cer24:1 (Bottom-right), increasing concentrations are associated with higher positive SHAP values.

We evaluated six machine learning algorithms to predict surrogate indices of IR using the circulating ceramide profile. The model exhibiting the highest overall discrimination was selected for further analysis, based on comprehensive performance metrics such as RMSE and MAE (**Supplementary Table 1****).** To assess the relative contribution of individual ceramide species, we applied SHAP analysis to the optimized model. The SHAP summary plot (**Figure 3A**) ranks feature according to their mean absolute SHAP values for the TyG. Higher concentrations of Cer24:0 (indicated by red points on the right) were associated with positive SHAP values, indicating that this species exerted the most significant upward influence on the predicted TyG score. Dependence plots for Cer16:0, Cer18:0, Cer24:0, and Cer24:1 (**Figure 3B**) demonstrated an approximately monotonic increase in SHAP value with rising serum concentrations for all species except Cer16:0. Similar concentration-dependent patterns were observed for TG/HDL-C and METS-IR (**Supplementary Figures 6**, **7**).

### 3.6 Development of a ceramide-based IR prediction model

IR was determined using a TyG threshold of 9 as the classification criterion. Initially, all candidate variables, incorporating demographic characteristics, clinical parameters, and the complete range of ceramides presented in **Table 1**, underwent analysis via the Least Absolute Shrinkage and Selection Operator (LASSO) regression to identify the most predictive features, as depicted in **Supplementary Figures 8**, **9**. This feature selection process yielded seven variables, which were subsequently incorporated into the final predictive model. The relative importance of these variables is presented in **Supplementary Figure 10****. A** systematic evaluation of the performance of six different modeling approaches was then conducted using ROC analysis and other performance metrics, as detailed in **Supplementary Table 2**. Of all the models tested, the GLM, implemented with a binomial distribution and logit link function, and incorporating ridge regularization (λ = 0.012), demonstrated superior predictive performance.

**Figure 4:**
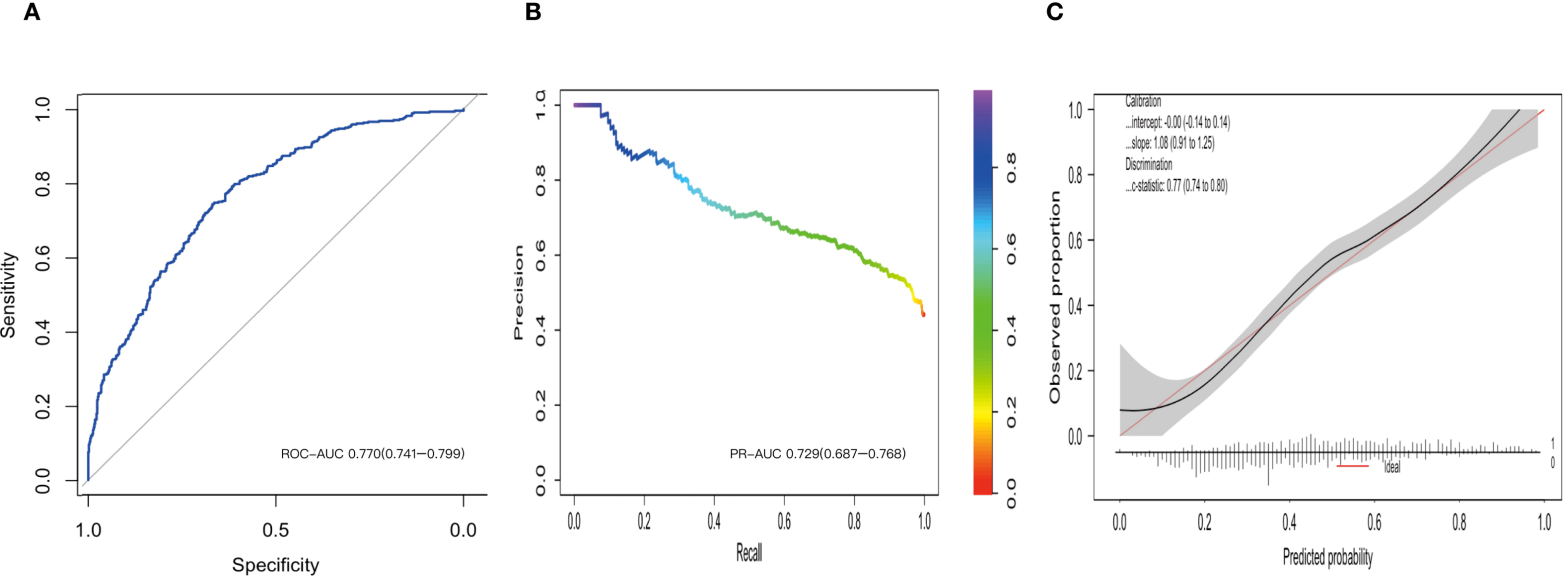
Discrimination, precision–recall performance, and calibration of the generalized linear model. **(A)** Receiver operating characteristic curve with area under the curve (ROC AUC). **(B)** Precision-Recall (PR) Curve: This curve illustrates the trade-off between Precision (Positive Predictive Value) and Recall (Sensitivity) across various classification thresholds. **(C)** Calibration Curve: This plot evaluates the concordance between the model’s predicted probabilities and the observed outcomes.

The ROC analysis demonstrated that the GLM possessed substantial discriminatory power, as indicated by ROC–AUC of 0.770 (95%CI, 0.741 to 0.799) (**Figure 4A**). This performance was further validated by the precision-recall curve, which showed an PR–AUC of 0.729 (95% CI, 0.687 to 0.768) (**Figure 4B**). The calibration assessment indicated a high level of agreement between predicted and observed event probabilities (**Figure 4C**), with a calibration intercept of -0.00 (95% CI, -0.14 to 0.14) and a calibration slope of 1.08 (95% CI, 0.91 to 1.25). At the F1-optimal threshold of 0.377, the overall classification error rate was 30.7%. The sensitivity was 79.8% (95% CI, 75.9% to 83.4%), while the specificity was 61.0% (95% CI, 56.8% to 65.0%). The corresponding confusion matrix is provided in **Table 3**. Internal validation through five-fold cross-validation confirmed the model’s robust performance, yielding a mean ROC–AUC of 0.764 ± 0.037 (**Supplementary Table 3**).

**Table 3:** Confusion matrix for IR prediction at F1-optimal threshold.

Actual\predicted	Non-IR	IR	Total
Non-IR	336	215	551
IR	88	348	436
Total	424	563	987

IR, Insulin Resistance.

**Figure 5:**
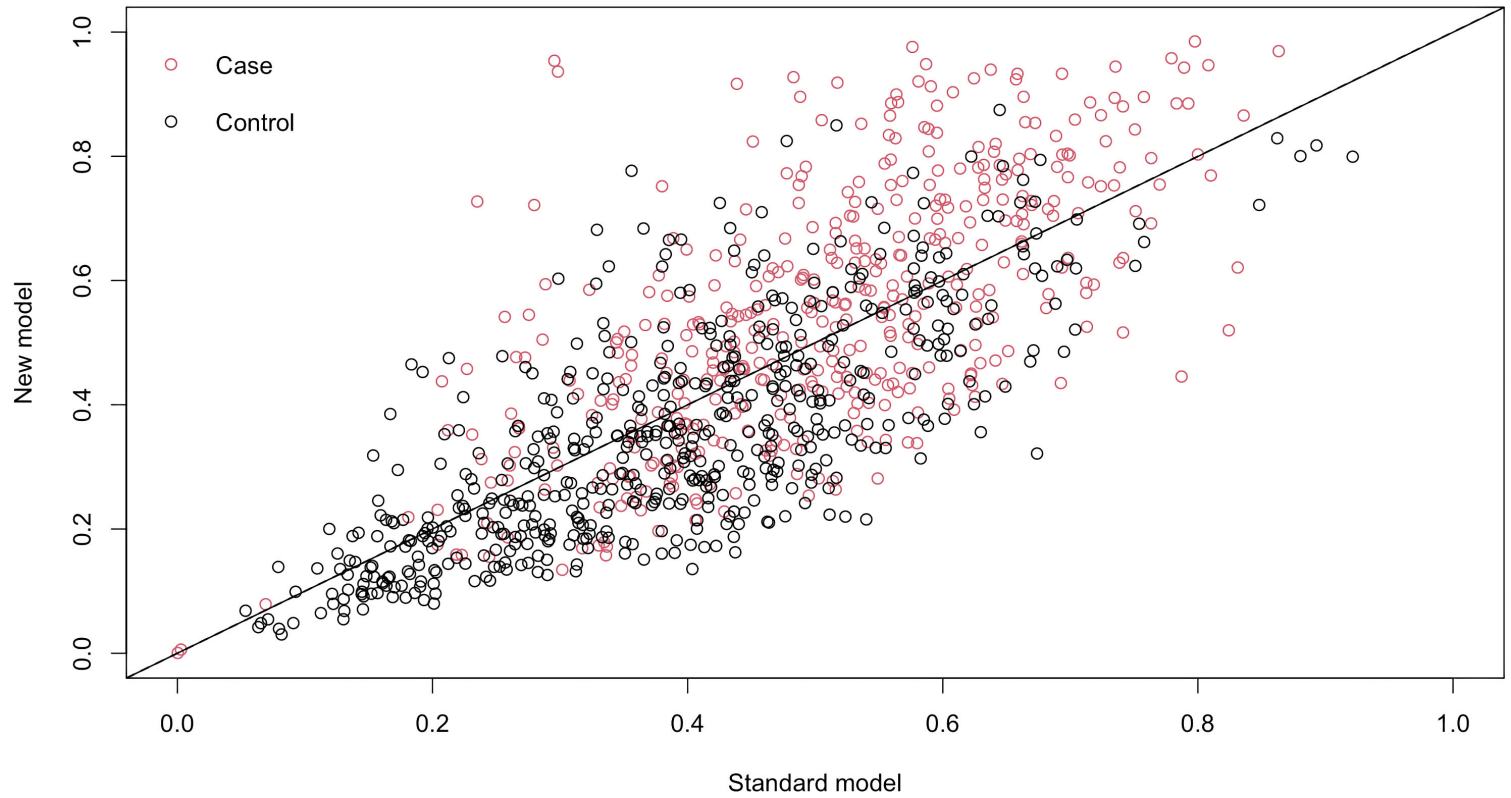
Reclassification scatter plots. The x-axis shows the predicted IR probability from the baseline GLM model with four non-ceramide LASSO-selected variables, while the y-axis shows the predicted IR probability from the full GLM model with all seven LASSO-selected variables, including ceramides.

To assess the additional predictive value provided by ceramides, we conducted a comparative analysis between a comprehensive GLM, which included all seven variables selected by the LASSO method, including ceramides, and a baseline GLM, which comprised only the four LASSO-selected variables excluding ceramides. The baseline model achieved a ROC-AUC of 0.713 (95%CI: 0.690 to 0.751). Incorporating ceramide variables into the full model led to a statistically significant overall category-free NRI of 0.54 (95% CI: 0.42 to 0.66). In particular, the event NRI was 0.23 (95% CI: 0.14 to 0.32), while the non-event NRI was 0.31 (95% CI: 0.23 to 0.39) (**Figure 5****).**

## 4 Discussion

In this multicenter cross-sectional study involving 987 Chinese adults with CAD undergoing coronary angiography, elevated plasma concentrations of specific ceramide species—most notably Cer24:0—were found to be associated with increased IR, as assessed by commonly used surrogate indices. These associations remained statistically significant following multivariable adjustment. Within a MGM, Cer24:0 demonstrated a direct conditional relationship with the TyG (partial correlation = 0.23), indicating a small-to-moderate association independent of measured covariates. RCS analyses supported concentration–response relationships, revealing that the association for Cer24:0 was approximately linear, whereas other ceramide species exhibited evidence of nonlinearity. Collectively, these findings suggest that profiling the ceramide spectrum—particularly Cer24:0—may provide complementary insights into IR.

In light of the current absence of a universally accepted gold standard for diagnosing IR in populations with CAD, this study operationally defines clinical insulin resistance as a TyG of ≥9.0. This threshold is informed by a synthesis of existing literature. Specifically, while the optimal TyG cutoffs for IR detection in the general population range from 8.0 to 8.5, patients with CAD exhibit considerably higher average TyG values due to inherent metabolic dysfunction (21, 22). Several prognostic studies underscore the clinical relevance of a TyG ≥9.0: for example, Jin et al. found that stable CAD patients in the highest TyG quartile (>9.17) had cardiovascular event rates of 20.3%, compared to 12.8-17.5% in lower quartiles (23). Similarly, critically ill cardiac patients with a TyG >9.32 demonstrated a 2.31-fold increase in cardiovascular mortality (24). Additionally, this threshold is pertinent to the Chinese population, as cross-national research reveals ethnic variations in TyG cutoffs (e.g., China: 9.72 *vs*. UK: 8.51), likely reflecting diverse metabolic profiles. A TyG of ≥9.0 corresponds to the median-to-upper tertile in well-established Chinese cohorts with CAD, thereby enabling effective stratification for the investigation of ceramide-insulin IR associations (25).

Ceramides, a class of sphingolipids, have been implicated in insulin resistance and the pathogenesis of type 2 diabetes (26). Various circulating ceramide species are associated with negative metabolic outcomes, with long-chain saturated ceramides (Cer16:0, Cer18:0) presenting the most robust evidence (27). However, certain very long-chain species (Cer24:0) also play a role in metabolic dysregulation (28). Ceramides interfere with insulin signaling by inhibiting Akt and disrupting the trafficking of GLUT4. The risk associated with ceramide chain length is supported by genetic data, although inconsistencies across studies may be attributed to variations in tissue compartments and outcome measures (29, 30). Notably, prospective cohort studies have not consistently demonstrated that plasma ceramides independently predict the onset of diabetes after adjusting for confounding variables, highlighting the necessity for further research in this area (31).

Our population-based analyses demonstrated a consistent positive association between Cer24:0 levels and the burden of IR, as indicated by the TyG and the TG/HDL-C ratio. These findings suggest a stage-dependent phenomenon: elevated Cer24:0 levels were associated with adverse IR phenotypes within our CAD cohort. However, event-based studies have consistently shown that relatively lower Cer24:0 levels (reflected in higher Cer16:0/Cer24:0 or Cer18:0/Cer24:0 ratios) are predictive of poorer cardiovascular outcomes, indicating potentially distinct biological roles across different stages of disease progression (32, 33). We propose that during early metabolic dysregulation, increasing Cer24:0 concentrations may serve as a “stress signal” indicative of lipid-centric IR onset. This hypothesis is supported by its strong association with the TyG and previous evidence linking Cer24:0 to early metabolic abnormalities. The CerS2-mediated response may represent an active metabolic adaptation to lipid excess (34, 35). In contrast, during the later and more advanced stages of disease, a relative decline in Cer24:0 levels could signify advanced metabolic and cardiovascular decompensation. This proposed stage-dependent alteration in the role of Cer24:0 might be attributable to several mechanisms: the prolonged elevation of ceramides may eventually lead to maladaptive effects; sustained VLC synthesis could reflect better-preserved cellular homeostasis (14, 18, 32).

Our research, which concentrates on proximal IR indices, seeks to identify early lipid-centric dysfunction. In contrast, ceramide predictors based on ratios in clinical event studies may more accurately represent the decline in adaptive biosynthetic capacity associated with advanced disease. This theoretical framework posits Cer24:0 as a dynamic biomarker, whose clinical significance is intricately linked to the stage of the disease and the context of measurement, thus potentially resolving the observed paradox. Future longitudinal and tissue-specific lipidomics studies will be essential to substantiate this stage-dependent hypothesis (18, 33).

We observed significant positive correlations between Cer24:0 and both the TyG and TG/HDL-C, but not with METS-IR. These findings suggest a more specific biological role for ceramides in lipid-centric metabolic dysfunction. Cer24:0, a very long-chain ceramide, is synthesized via *de novo* pathways involving saturated fatty acids through the actions of serine palmitoyltransferase (SPT) and ceramide synthase 2 (CerS2) (36). Cer24:0 tends to accumulate under conditions characterized by elevated triglycerides and dysregulated glucose metabolism, as indicated by increased TyG levels. Mechanistically, Cer24:0 may inhibit Akt-dependent insulin signaling, promote triglyceride storage through the activation of sterol regulatory element-binding proteins (SREBP), and has been implicated in reducing HDL-C mediated protection (37, 38). This establishes a mechanistic foundation for the link between ceramide accumulation and the lipid-centric dysregulation represented by TyG and TG/HDL-C. In contrast, METS-IR incorporates BMI as a variable, offering a more holistic evaluation that extends beyond solely lipid-mediated mechanisms. Consequently, the differential association between Cer24:0 and both the TyG and TG/HDL-C aligns with the intimate biological connection between ceramide-induced lipotoxicity and lipid-centric insulin resistance. Meanwhile, the broader metabolic framework of METS-IR might account for the diminished direct association observed in this context.

The SHAP analysis indicated that elevated concentrations of Cer24:0 were associated with increased predicted TyG values, as evidenced by positive SHAP values, highlighting its significant contribution to the predictive model. These findings align directionally with the direct conditional association between Cer24:0 and TyG observed in the MGM. Notably, the DML estimates corroborated these results, demonstrating positive average association along the TyG and TG/HDL-C axes, while showing no statistically significant adjusted association for METS-IR. Collectively, these complementary methodologies—focusing on predictive contributions (SHAP), conditional associations (MGM), and average adjusted association under explicit assumptions (DML)—present a consistent pattern that supports a lipid-centric insulin-resistance axis centered on Cer24:0.

In the MGM analysis, we identified a partial correlation coefficient of 0.23 between Cer24:0 and the TyG. Although this standardized association exhibited moderate statistical strength, the observed variations in Cer24:0 levels, which may influence TyG, underscore the potential significance of Cer24:0 in clinical evaluations. Specifically, the APE of 0.459 for Cer24:0 on TyG, as estimated by the DML model, suggests that each one-unit increase in the natural logarithm of Cer24:0 is associated with an average increase of 0.459 units in TyG. Consequently, an alteration in Cer24:0 concentration from the 25th percentile (1400 ng/mL) to the 75th percentile (2270 ng/mL) could result in an approximate 0.22 unit increase in the predicted TyG.

This numerical difference has potential clinical implications, as it may elucidate how variations in Cer24:0 levels could alter patients’ metabolic risk classifications across established clinical risk thresholds. For example, the median TyG in our study cohort was 8.9, typically indicative of patients at intermediate metabolic risk. An independent increase of approximately 0.22 units, potentially associated with elevated Cer24:0 levels, could elevate the TyG for these patients to approximately 9.12. Importantly, this projected TyG value closely aligns with high-risk thresholds identified in extensive coronary artery disease cohort studies, such as those by Jin et al., which define a TyG greater than 9.17 as a high-risk threshold. These thresholds are frequently linked to a notable increase in the risk of major adverse cardiovascular events, with relevant research indicating a relative risk increase of approximately 60% (23).

The “tipping point” phenomenon implies that Cer24:0 levels could function as an auxiliary biomarker for the enhanced identification of individuals at significant metabolic risk who may benefit from intensified intervention strategies. While the TyG is commonly employed as an accessible and cost-effective surrogate biomarker, our findings suggest that Cer24:0 may provide more nuanced risk stratification beyond the classifications based on the TyG. Specifically, among patients with comparable and critical TyG values, Cer24:0 profiling may facilitate the differentiation of individuals with an elevated susceptibility to metabolic dysregulation. This differentiation could, in turn, offer more precise risk stratification information to inform decisions regarding the use of potent metabolic agents, such as glucagon-like peptide-1 receptor agonists, thereby enhancing clinical decision-making.

In addition to its potential application in suggesting individual patient risk, the results of our predictive model offer quantitative evidence for the added value of this biomarker at the population level. The ROC-AUC of the model increased from 0.71 in the baseline model to 0.77 with the incorporation of ceramides. Moreover, the NRI demonstrated that the inclusion of ceramide variables resulted in a statistically significant overall category-free NRI of 0.54 (Event NRI: 0.23; Non-Event NRI: 0.31). Clinically, these findings suggest that Cer24:0 profiling may enhance the precision of IR phenotype classification. Specifically, it may assist in identifying patients with more severe IR profiles who may benefit from targeted metabolic interventions and in screening patients who might otherwise undergo unnecessary investigations or treatments for IR.

Several limitations warrant consideration in this study. First, due to its cross-sectional design, we cannot establish a temporal sequence between ceramide levels and IR status. This inherently limits our ability to infer causality, and issues such as reverse causation and residual confounding cannot be entirely ruled out. Second, IR was assessed using surrogate indices rather than the hyperinsulinemia–euglycemic clamp, which is considered the gold standard. Although these surrogates are widely validated and practical, they may lead to misclassification of IR and may vary in their construct coverage. Third, the ceramide panel employed in this study was limited to the four most prevalent and clinically validated plasma species (Cer16:0, Cer18:0, Cer24:0, Cer24:1). Adopting a more extensive sphingolipidomic profiling methodology, which includes dihydroceramides (e.g., dhCer22:2) and ceramide ratios (e.g., Cer16:0/Cer24:0), could enhance mechanistic understanding and improve predictive accuracy. This approach would allow for the identification of upstream metabolic dysregulation and the equilibrium between deleterious and protective ceramide species. Fourth, despite the implementation of multivariable and network-based methodologies in this investigation, residual confounding presents a notable limitation. Specifically, several critical confounding variables, including medication usage (e.g., statins and antidiabetic agents such as insulin, metformin, and SGLT2 inhibitors), dietary patterns, and visceral adiposity, were not thoroughly measured or adequately adjusted for in the analysis. Given their well-documented and significant impact on both ceramide levels and indices of insulin resistance, this limitation may contribute to residual confounding in the observed associations, potentially compromising the accuracy of the study’s findings. Future research should prioritize the collection of comprehensive data on these confounders and implement appropriate adjustments in the analytical framework. Finally, this study was conducted exclusively on Chinese patients with CAD undergoing coronary evaluation. Consequently, the findings may not be generalizable to non-CAD populations, individuals of other ethnic backgrounds—considering ethnic-specific metabolic variations—or primary prevention contexts. Therefore, validation in diverse cohorts is necessary to establish broader generalizability.

## 5 Conclusion

Elevated concentrations of specific ceramides, notably Cer24:0, exhibit a significant correlation with IR as assessed through commonly employed surrogate markers. Analyses utilizing MGM reveal a direct conditional association between Cer24:0 and the TyG. SHAP analysis indicates that, in models predicting TyG as a surrogate marker for IR, Cer24:0 exhibits the highest marginal significance among ceramide molecules. The ceramide-based predictive model demonstrates moderate accuracy in identifying IR, defined as TyG≥9. These findings suggest that evaluating ceramide profiles, particularly Cer24:0, may enhance understanding of metabolic risk in patients with CAD.

## Data Availability

The raw data supporting the conclusions of this article will be made available by the authors, without undue reservation.

## References

[1] Feigin VL Stark BA Johnson CO Roth GA Bisignano C Abady GG . Global, regional, and national burden of stroke and its risk factors, 1990–2019: a systematic analysis for the Global Burden of Disease Study 2019. Lancet Neurol. (2021) 20:795–820. doi: 10.1016/S1474-4422(21)00252-0, PMID: 34487721 PMC8443449

[2] Garcia-Carretero R Vazquez-Gomez O Gil-Prieto R Gil-de-Miguel A . Insulin resistance is a cardiovascular risk factor in hypertensive adults without type 2 diabetes mellitus. Wien Klin Wochenschr. (2024) 136:101–9. doi: 10.1007/s00508-023-02278-1, PMID: 37814058

[3] Kassi E Pervanidou P Kaltsas G Chrousos G . Metabolic syndrome: definitions and controversies. BMC Med. (2011) 9:48. doi: 10.1186/1741-7015-9-48, PMID: 21542944 PMC3115896

[4] El-Amouri S Karakashian A Bieberich E Nikolova-Karakashian M . Regulated translocation of neutral sphingomyelinase-2 to the plasma membrane drives insulin resistance in steatotic hepatocytes. J Lipid Res. (2023) 64:100435. doi: 10.1016/j.jlr.2023.100435, PMID: 37640282 PMC10550728

[5] Hilvo M Vasile VC Donato LJ Hurme R Laaksonen R . Ceramides and ceramide scores: clinical applications for cardiometabolic risk stratification. Front Endocrinol (Lausanne). (2020) 11:570628. doi: 10.3389/fendo.2020.570628, PMID: 33133018 PMC7550651

[6] He X Schuchman EH . Ceramide and ischemia/reperfusion injury. J Lipids. (2018) 2018:1–11. doi: 10.1155/2018/3646725, PMID: 29610685 PMC5828470

[7] Field BC Gordillo R Scherer PE . The role of ceramides in diabetes and cardiovascular disease regulation of ceramides by adipokines. Front Endocrinol (Lausanne). (2020) 11:569250. doi: 10.3389/fendo.2020.569250, PMID: 33133017 PMC7564167

[8] Otowa-Suematsu N Sakaguchi K Kaneko A Ito J Morita Y Miura H . Relation of cardiac function to insulin resistance as evaluated by hyperinsulinemic-euglycemic clamp analysis in individuals with type 2 diabetes. J Diabetes Investig. (2021) 12:2197–202. doi: 10.1111/jdi.13608, PMID: 34081831 PMC8668073

[9] Zhou Z Liu Q Zheng M Zuo Z Zhang G Shi R . Comparative study on the predictive value of TG/HDL-C, TyG and TyG-BMI indices for 5-year mortality in critically ill patients with chronic heart failure: a retrospective study. Cardiovasc Diabetol. (2024) 23:213. doi: 10.1186/s12933-024-02308-w, PMID: 38902757 PMC11191322

[10] Tsai K-Z Chu C-C Huang W-C Sui X Lavie CJ Lin G-M . Prediction of various insulin resistance indices for the risk of hypertension among military young adults: the CHIEF cohort study, 2014–2020. Cardiovasc Diabetol. (2024) 23:141. doi: 10.1186/s12933-024-02229-8, PMID: 38664804 PMC11046748

[11] Jiang W Ogretmen B . Autophagy paradox and ceramide. Biochim Biophys Acta (BBA) - Mol Cell Biol Lipids. (2014) 1841:783–92. doi: 10.1016/j.bbalip.2013.09.005, PMID: 24055889 PMC3960371

[12] Chaurasia B Summers SA . Ceramides in metabolism: key lipotoxic players. Annu Rev Physiol. (2021) 83:303–30. doi: 10.1146/annurev-physiol-031620-093815, PMID: 33158378 PMC7905841

[13] Delcheva G Stefanova K Stankova T . Ceramides—Emerging biomarkers of lipotoxicity in obesity, diabetes, cardiovascular diseases, and inflammation. Diseases. (2024) 12:195. doi: 10.3390/diseases12090195, PMID: 39329864 PMC11443555

[14] Cresci S Zhang R Yang Q Duncan MS Xanthakis V Jiang X . Genetic architecture of circulating very-long-chain (C24:0 and C22:0) ceramide concentrations. J Lipid Atheroscler. (2020) 9:172–83. doi: 10.12997/jla.2020.9.1.172, PMID: 32489964 PMC7266332

[15] Kasumov T Huang H Chung Y-M Zhang R McCullough AJ Kirwan JP . Quantification of ceramide species in biological samples by liquid chromatography electrospray ionization tandem mass spectrometry. Anal Biochem. (2010) 401:154–61. doi: 10.1016/j.ab.2010.02.023, PMID: 20178771 PMC2872137

[16] Park J-W Park W-J Futerman AH . Ceramide synthases as potential targets for therapeutic intervention in human diseases. Biochim Biophys Acta. (2014) 1841:671–81. doi: 10.1016/j.bbalip.2013.08.019, PMID: 24021978

[17] Tidhar R Zelnik ID Volpert G Ben-Dor S Kelly S Merrill AH . Eleven residues determine the acyl chain specificity of ceramide synthases. J Biol Chem. (2018) 293:9912–21. doi: 10.1074/jbc.RA118.001936, PMID: 29632068 PMC6016465

[18] Nwabuo CC Duncan M Xanthakis V Peterson LR Mitchell GF McManus D . Association of circulating ceramides with cardiac structure and function in the community: the framingham heart study. J Am Heart Assoc. (2019) 8:e013050. doi: 10.1161/JAHA.119.013050, PMID: 31549564 PMC6806035

[19] Peterson LR Xanthakis V Duncan MS Gross S Friedrich N Völzke H . Ceramide remodeling and risk of cardiovascular events and mortality. J Am Heart Assoc. (2018) 7:e007931. doi: 10.1161/JAHA.117.007931, PMID: 29728014 PMC6015315

[20] Chernozhukov V Cinelli C Newey W Sharma A Syrgkanis V . Long story short: omitted variable bias in causal machine learning. National Bureau of Economic Research (2024) 30302. doi: 10.3386/w30302, PMID: 34419315

[21] Wang L Cong H-L Zhang J-X Hu Y-C Wei A Zhang Y-Y . Triglyceride-glucose index predicts adverse cardiovascular events in patients with diabetes and acute coronary syndrome. Cardiovasc Diabetol. (2020) 19:80. doi: 10.1186/s12933-020-01054-z, PMID: 32534586 PMC7293784

[22] Kurniawan LB . Triglyceride-glucose index as A biomarker of insulin resistance, diabetes mellitus, metabolic syndrome, and cardiovascular disease: A review. EJIFCC. (2024) 35:44–51. 38706737 PMC11063788

[23] Jin J-L Cao Y-X Wu L-G You X-D Guo Y-L Wu N-Q . Triglyceride glucose index for predicting cardiovascular outcomes in patients with coronary artery disease. J Thorac Dis. (2018) 10:6137–46. doi: 10.21037/jtd.2018.10.79, PMID: 30622785 PMC6297409

[24] Feng B Zhao Y Xu W Meng X Li Y Xia C . Triglyceride-glucose index as a novel prognostic biomarker for coronary artery disease: evidence from a large-scale prospective cohort study. Front Endocrinol (Lausanne). (2025) 16:1653948. doi: 10.3389/fendo.2025.1653948, PMID: 41019316 PMC12460115

[25] Fang C Peng N Cheng J Zhang X Gu W Zhu Z . The association between TyG index and cardiovascular mortality is modified by antidiabetic or lipid-lowering agent: a prospective cohort study. Cardiovasc Diabetol. (2025) 24:65. doi: 10.1186/s12933-025-02620-z, PMID: 39920712 PMC11806668

[26] Galadari S Rahman A Pallichankandy S Galadari A Thayyullathil F . Role of ceramide in diabetes mellitus: evidence and mechanisms. Lipids Health Dis. (2013) 12:98. doi: 10.1186/1476-511X-12-98, PMID: 23835113 PMC3716967

[27] Fretts AM Jensen PN Hoofnagle AN McKnight B Howard BV Umans J . Plasma ceramides containing saturated fatty acids are associated with risk of type 2 diabetes. J Lipid Res. (2021) 62:100119. doi: 10.1016/j.jlr.2021.100119, PMID: 34555371 PMC8517199

[28] Jiang H Hsu F-F Farmer MS Peterson LR Schaffer JE Ory DS . Development and validation of LC-MS/MS method for determination of very long acyl chain (C22:0 and C24:0) ceramides in human plasma. Anal Bioanal Chem. (2013) 405:7357–65. doi: 10.1007/s00216-013-7166-9, PMID: 23857140 PMC3766747

[29] Hage Hassan R Pacheco de Sousa AC Mahfouz R Hainault I Blachnio-Zabielska A Bourron O . Sustained action of ceramide on the insulin signaling pathway in muscle cells: IMPLICATION OF THE DOUBLE-STRANDED RNA-ACTIVATED PROTEIN KINASE. J Biol Chem. (2016) 291:3019–29. doi: 10.1074/jbc.M115.686949, PMID: 26698173 PMC4742763

[30] Mahfouz R Khoury R Blachnio-Zabielska A Turban S Loiseau N Lipina C . Characterising the inhibitory actions of ceramide upon insulin signaling in different skeletal muscle cell models: a mechanistic insight. PloS One. (2014) 9:e101865. doi: 10.1371/journal.pone.0101865, PMID: 25058613 PMC4109934

[31] Neeland IJ Singh S McGuire DK Vega GL Roddy T Reilly DF . Relation of plasma ceramides to visceral adiposity, insulin resistance and the development of type 2 diabetes mellitus: the Dallas Heart Study. Diabetologia. (2018) 61:2570–9. doi: 10.1007/s00125-018-4720-1, PMID: 30159588 PMC6219923

[32] Mathews AT Famodu OA Olfert MD Murray PJ Cuff CF Downes MT . Efficacy of nutritional interventions to lower circulating ceramides in young adults: FRUVEDomic pilot study. Physiol Rep. (2017) 5:e13329. doi: 10.14814/phy2.13329, PMID: 28694327 PMC5506522

[33] Bergman BC Brozinick JT Strauss A Bacon S Kerege A Bui HH . Serum sphingolipids: relationships to insulin sensitivity and changes with exercise in humans. Am J Physiol Endocrinol Metab. (2015) 309:E398–408. doi: 10.1152/ajpendo.00134.2015, PMID: 26126684 PMC4537923

[34] Hilvo M Jylhä A Lääperi M Jousilahti P Laaksonen R . Absolute and relative risk prediction in cardiovascular primary prevention with a modified SCORE chart incorporating ceramide-phospholipid risk score and diabetes mellitus. Eur Heart J Open. (2021) 1:oeab010. doi: 10.1093/ehjopen/oeab010, PMID: 35919880 PMC9242040

[35] Broberg O Weismann CG Øra I Wiebe T Laaksonen R Liuba P . Ceramides: a potential cardiovascular biomarker in young adult childhood cancer survivors? Eur Heart J Open. (2024) 4:oeae026. doi: 10.1093/ehjopen/oeae026, PMID: 38659666 PMC11042783

[36] Park T-S Hu Y Noh H-L Drosatos K Okajima K Buchanan J . Ceramide is a cardiotoxin in lipotoxic cardiomyopathy. J Lipid Res. (2008) 49:2101–12. doi: 10.1194/jlr.M800147-JLR200, PMID: 18515784 PMC2533410

[37] Shu H Peng Y Hang W Li N Zhou N Wang DW . Emerging roles of ceramide in cardiovascular diseases. Aging Dis. (2022) 13:232. doi: 10.14336/AD.2021.0710, PMID: 35111371 PMC8782558

[38] Tippetts TS Holland WL Summers SA . Cholesterol - the devil you know; ceramide - the devil you don’t. Trends Pharmacol Sci. (2021) 42:1082–95. doi: 10.1016/j.tips.2021.10.001, PMID: 34750017 PMC8595778

