# Supplementary material for: Plasma ceramide Cer24:0 and insulin resistance: associations with TyG and TG/HDL-C in a multicenter study of coronary artery disease cohorts
Source: Front Endocrinol (Lausanne). 2026 Feb 26;17:1777380. doi: 10.3389/fendo.2026.1777380 (PMC12979231; doi:10.3389/fendo.2026.1777380)
Supplement: Supplementary file 1 [file DataSheet1.doc]

**Supplementary Materials for Plasma Ceramide Cer24:0 and Insulin Resistance: Associations with TyG and TG/HDL-C in a Multicenter Study of Coronary Artery Disease Cohorts**

Contents:

Supplementary Fig.1 Spearman correlation heatmap of ceramide species and insulin resistance markers.

Supplementary Fig.2. Forest plot of mixed graphical model edge-weight estimates involving TyG with 95% confidence intervals.

Supplementary Fig.3. Forest plot of mixed graphical model edge-weight estimates involving TG/HDL-C with 95% confidence intervals.

Supplementary Fig.4. Forest plot of mixed graphical model edge-weight estimates involving METS-IR with 95% confidence intervals

Supplementary Fig.5 Restricted cubic spline (RCS) analyses showing univariable associations between plasma ceramide concentrations and indices of insulin resistance.

Supplementary Fig.6 SHAP analysis for prediction of the TG/HDL-C.

Supplementary Fig.7. SHAP analysis for prediction of the METS-IR.

Supplementary Fig.8 LASSO coefficient paths.

Supplementary Fig.9 Cross validated mean squared error (MSE) for the least absolute shrinkage and selection operator (LASSO) as a function of log(λ).

Supplementary Fig.10 Relative importance of predictors in the final generalized linear model for insulin resistance.

Supplementary Table 1. Performance metrics of machine learning models for IR prediction using serum ceramide profiles.

Supplementary Table 2. Machine learning model performance for ceramide-based IR prediction.

Supplementary Table 3. Performance metrics of the model in 5-fold cross-validation


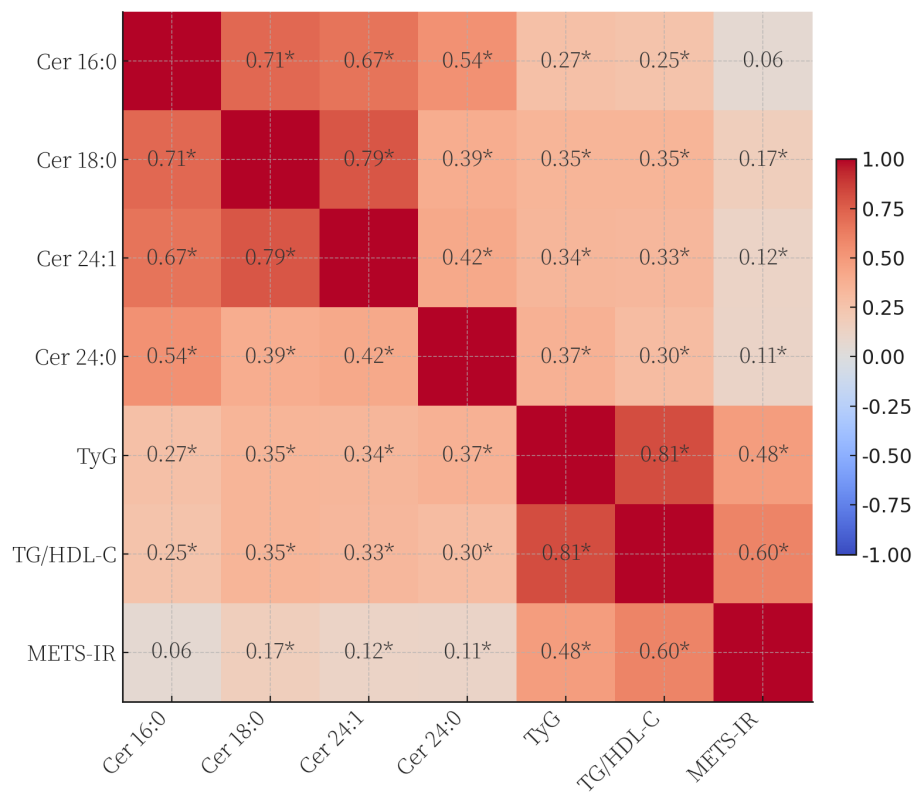


**Supplementary Fig.1 Spearman correlation heatmap of ceramide species and insulin resistance markers.** TyG: triglyceride-glucose index; METS-IR: metabolic score for insulin resistance; TG/HDL-C: triglyceride to high-density lipoprotein cholesterol ratio. * P < 0.05


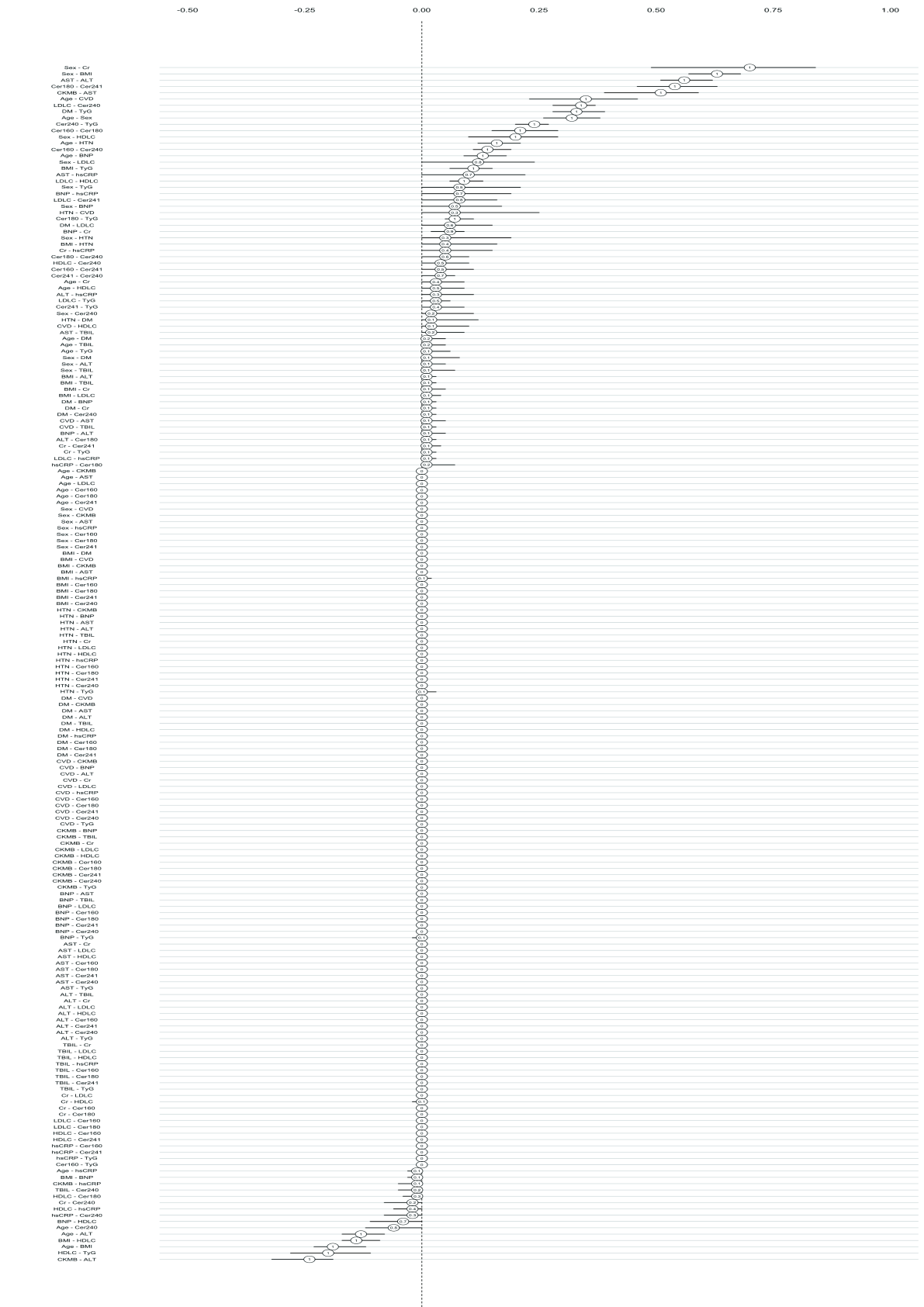


**Supplementary Fig.2. Forest plot of mixed graphical model edge-weight estimates involving TyG with 95% confidence intervals.**

Abbreviations: ALT: alanine aminotransferase; AST: aspartate aminotransferase; BMI: body mass index; BNP: B-type natriuretic peptide; Cer: ceramide; CK-MB: creatine kinase MB; Cr: creatinine; CVD: cerebrovascular disease; DM: diabetes mellitus; HDL-C: high-density lipoprotein cholesterol; hsCRP: high-sensitivity C-reactive protein; HTN: hypertension; LDL-C: low-density lipoprotein cholesterol; TBIL: total bilirubin; TyG: triglyceride–glucose index.

**
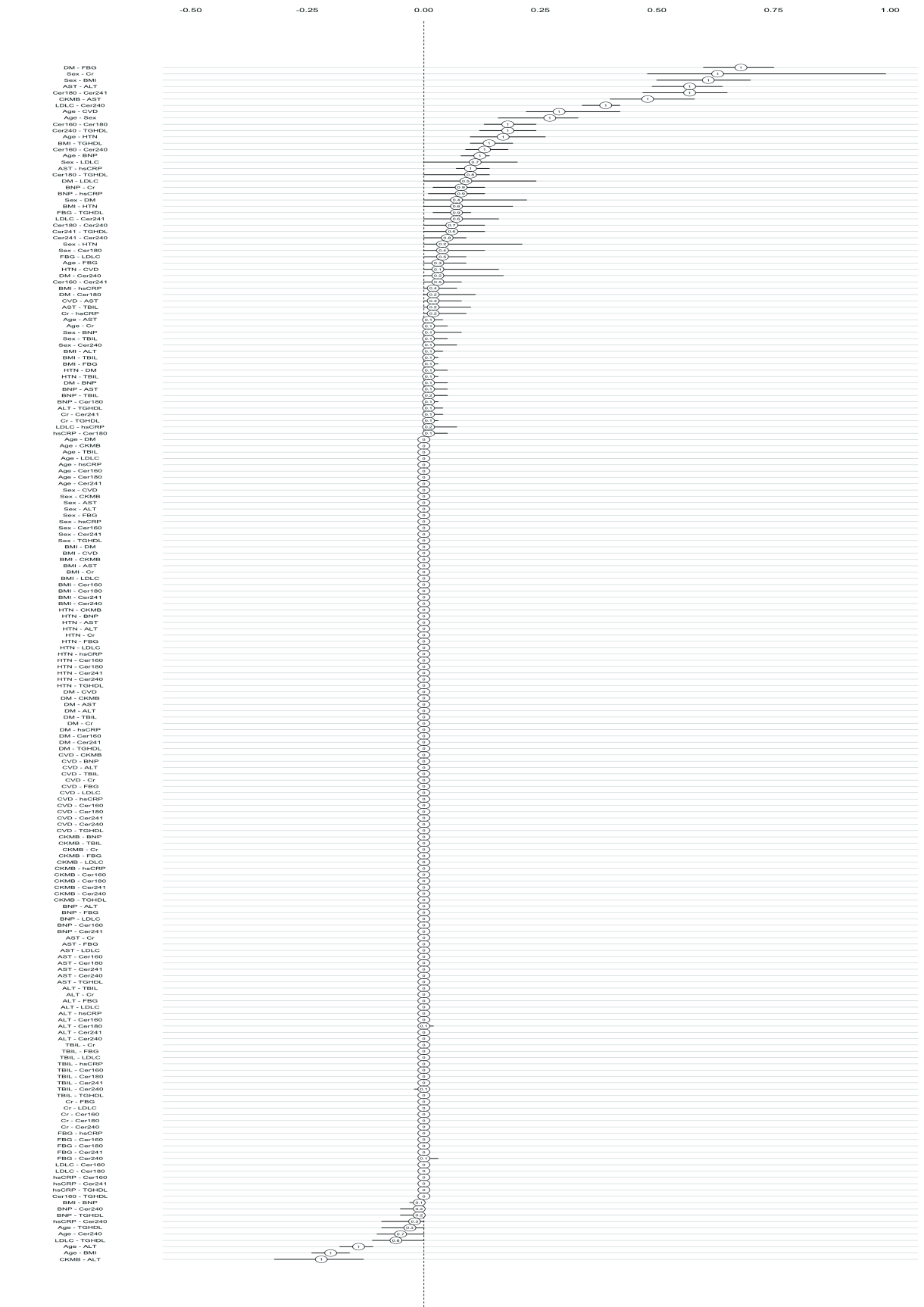
Supplementary Fig.3. Forest plot of mixed graphical model edge-weight estimates involving TG/HDL-C with 95% confidence intervals.**

ALT: alanine aminotransferase; AST: aspartate aminotransferase; BMI: body mass index; BNP: B-type natriuretic peptide; Cer: ceramide; CK-MB: creatine kinase MB; Cr: creatinine; CVD: cerebrovascular disease; DM: diabetes mellitus; FBG: fasting blood glucose; HDL-C: high-density lipoprotein cholesterol; hsCRP: high-sensitivity C-reactive protein; HTN: hypertension; LDL-C: low-density lipoprotein cholesterol; METS-IR: metabolic score for insulin resistance; TBIL: total bilirubin; TG/HDL: triglyceride-to-HDL cholesterol ratio.


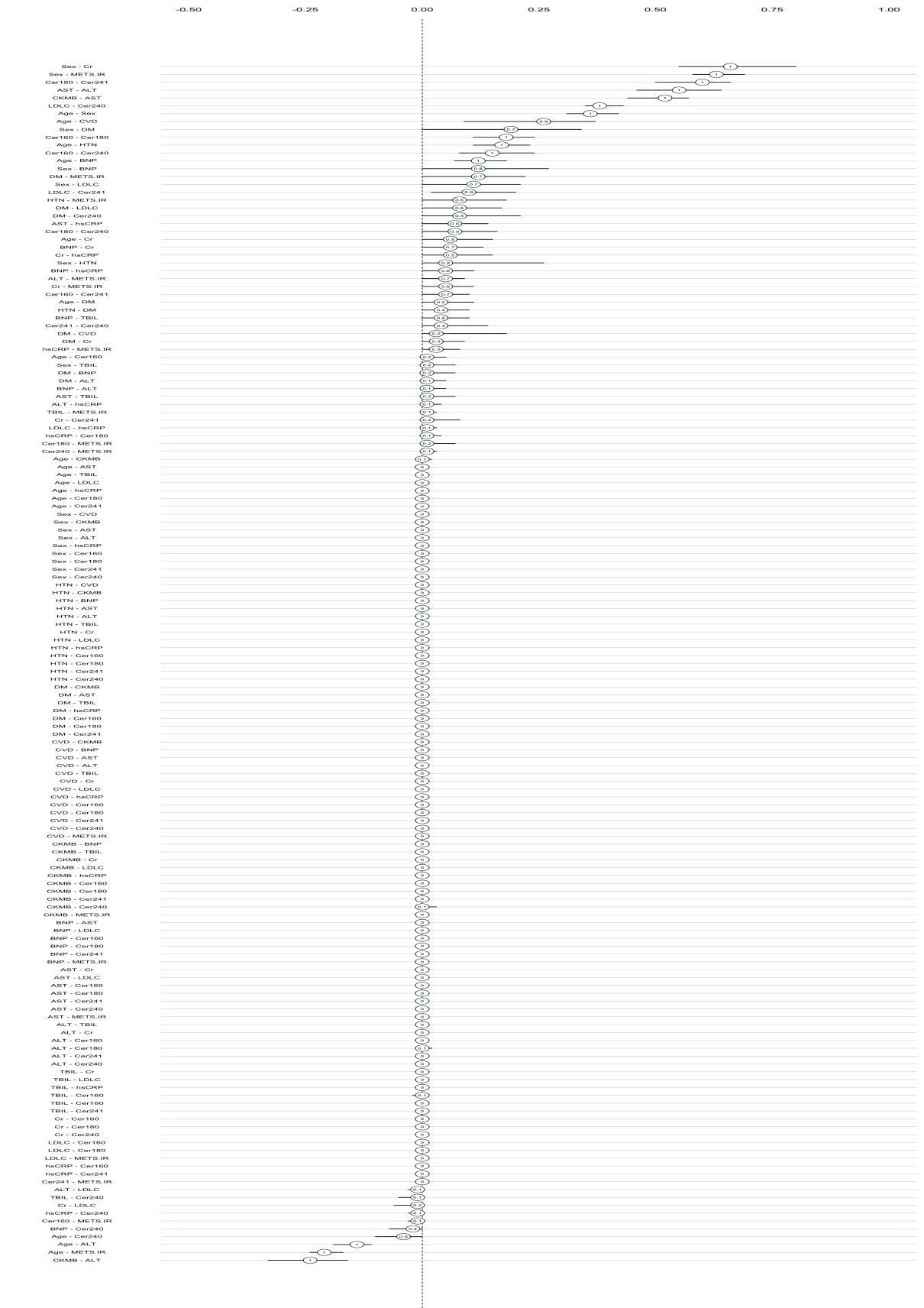


**Supplementary Fig.4. Forest plot of mixed graphical model edge-weight estimates involving METS-IR with 95% confidence intervals**

ALT: alanine aminotransferase; AST: aspartate aminotransferase; BNP: B-type natriuretic peptide; Cer: ceramide; CK-MB: creatine kinase MB; Cr: creatinine; CVD: cerebrovascular disease; DM: diabetes mellitus; hsCRP: high-sensitivity C-reactive protein; HTN: hypertension; LDL-C: low-density lipoprotein cholesterol; METS-IR: metabolic score for insulin resistance; TBIL: total bilirubin.


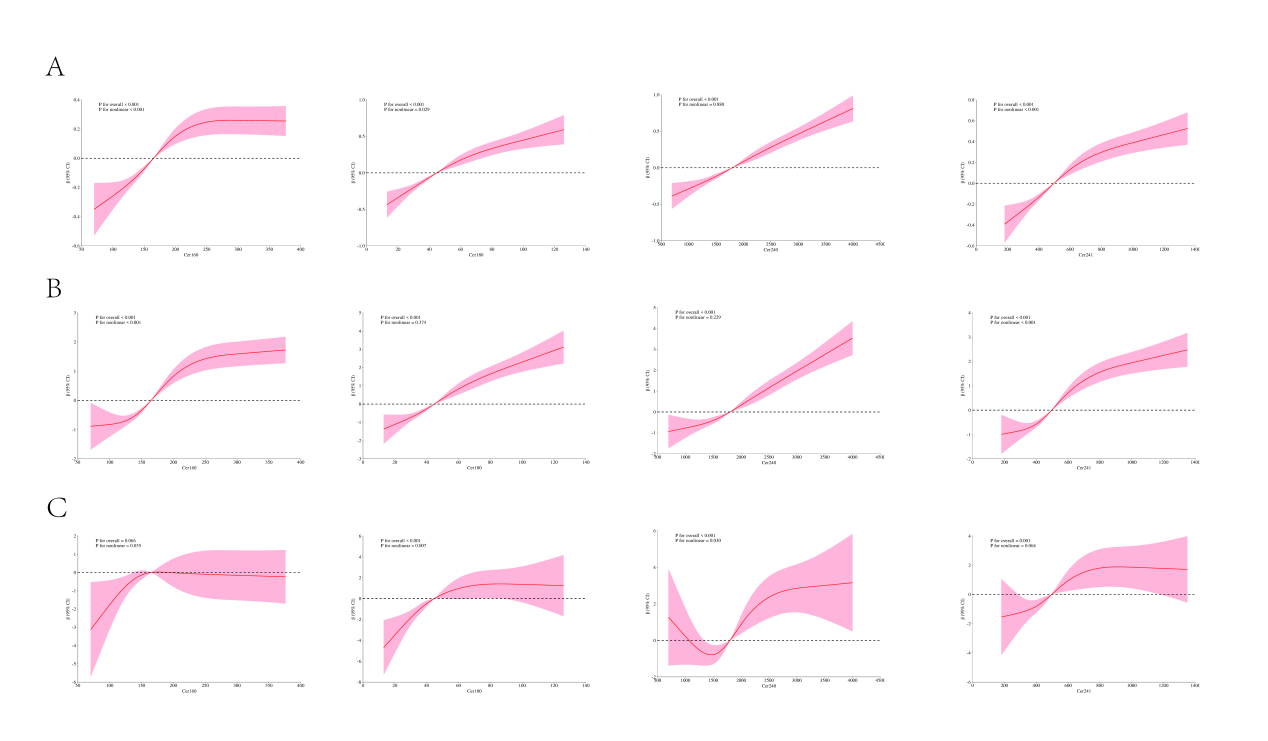


**Supplementary Fig.5 Restricted cubic spline (RCS) analyses showing univariable associations between plasma ceramide concentrations and indices of insulin resistance.** A: TyG; B: TG/HDL-C; C: METS-IR. METS‑IR: metabolic score for insulin resistance; TG/HDL‑C: triglyceride‑to–HDL cholesterol ratio; TyG: triglyceride–glucose index.

| **Supplementary Table 1. Performance metrics of machine learning models for IR prediction using serum ceramide profiles** | | | | | |
| --- | --- | --- | --- | --- | --- |
| **A: TyG** | | | | | |
| Model | Rmse | Mse | Mae | Rmsle | Mean ResidualD eviance |
| GBM | 0.524 | 0.275 | 0.411 | 0.052 | 0.275 |
| DRF | 0.528 | 0.279 | 0.417 | 0.052 | 0.279 |
| XGBoost | 0.541 | 0.293 | 0.430 | 0.054 | 0.293 |
| DeepLearning | 0.543 | 0.295 | 0.425 | 0.054 | 0.295 |
| XRT | 0.544 | 0.296 | 0.432 | 0.054 | 0.296 |
| GLM | 0.544 | 0.296 | 0.423 | 0.054 | 0.296 |
| **B: TG/HDL-C** | | | | | |
| Model | Rmse | Mse | Mae | Rmsle | Mean ResidualD eviance |
| DeepLearning | 2.474 | 6.123 | 1.746 | 0.434 | 6.123 |
| DRF | 2.519 | 6.347 | 1.712 | 0.423 | 6.347 |
| XRT | 2.520 | 6.352 | 1.722 | 0.424 | 6.352 |
| GBM | 2.531 | 6.408 | 1.707 | 0.422 | 6.408 |
| GLM | 2.588 | 6.699 | 1.737 | 0.429 | 6.699 |
| XGBoost | 2.704 | 7.309 | 1.852 | 0.457 | 7.309 |
| **C: METS-IR** | | | | | |
| Model | Rmse | Mse | Mae | Rmsle | Mean ResidualD eviance |
| GBM | 7.419 | 55.047 | 5.737 | 0.182 | 55.047 |
| XGBoost | 7.678 | 58.951 | 5.962 | 0.188 | 58.951 |
| DRF | 7.770 | 60.372 | 6.089 | 0.191 | 60.372 |
| XRT | 7.870 | 61.941 | 6.178 | 0.193 | 61.941 |
| DeepLearning | 7.913 | 62.617 | 6.153 | 0.193 | 62.617 |
| GLM | 7.924 | 62.798 | 6.132 | 0.192 | 62.798 |
| DRF, Distributed Random Forest; GBM, Gradient Boosting Machine; GLM, Generalized Linear Model; HDL-c, high-density lipoprotein cholesterol; IR, insulin resistance; MAE, mean absolute error; Mean Residual Deviance, mean residual deviance; METS-IR, Metabolic Score for Insulin Resistance; MSE, mean square error; RMSE, root mean square error; RMSLE, root mean squared logarithmic error; TG, triglyceride; TG/HDL‑C: triglyceride‑to–HDL cholesterol ratio; TyG, triglyceride-glucose index; XGBoost, Extreme Gradient Boosting; XRT, Extremely Randomized Trees. | | | | | |


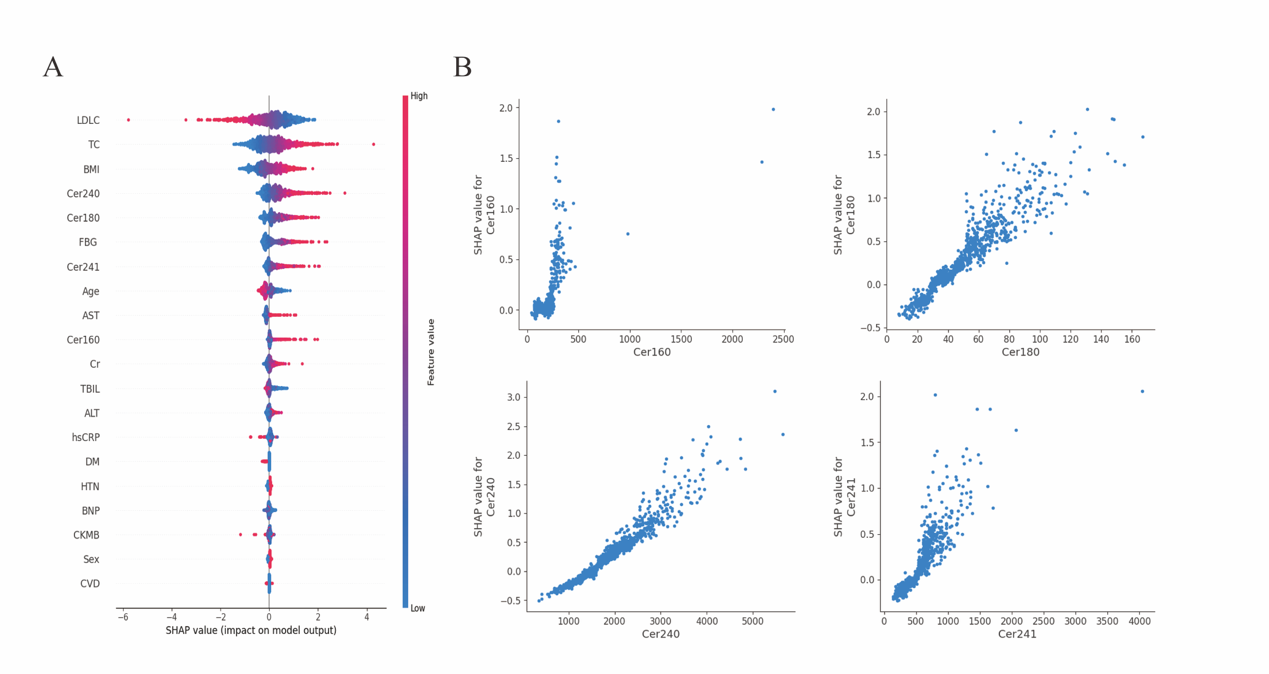


**Supplementary Fig.6 SHAP analysis for prediction of the TG/HDL-C.**

A: SHAP summary plot ranking features by mean absolute SHAP value. The x-axis shows SHAP values (impact on model output) and the y-axis lists features; point color encodes the feature value for each observation. Positive SHAP values indicate a higher predicted TG/HDL-C index. B: SHAP dependence plots for Cer16:0, Cer18:0, Cer24:0, and Cer24:1, showing feature value (x-axis) versus SHAP value (y-axis); each point represents one participant. Cer, ceramide; SHAP, Shapley Additive Explanations; TG/HDL‑C: triglyceride‑to–HDL cholesterol ratio


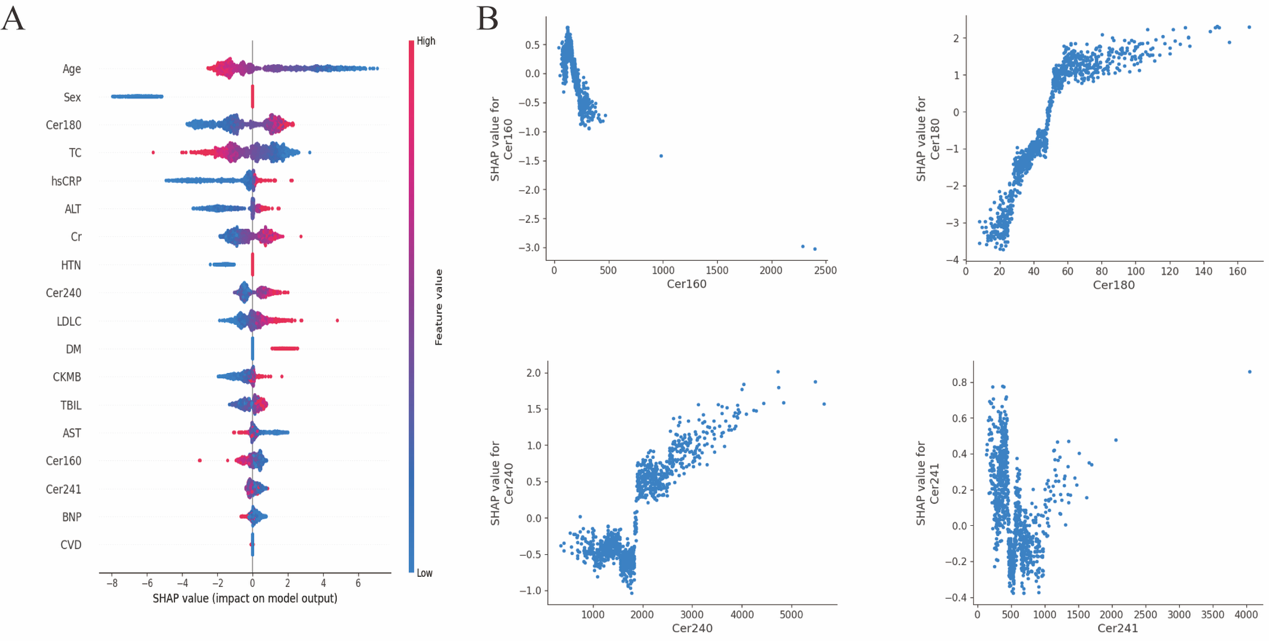


**Supplementary Fig.7. SHAP analysis for prediction of the METS-IR.**

A: SHAP summary plot ranking features by mean absolute SHAP value. The x-axis shows SHAP values (impact on model output) and the y-axis lists features; point color encodes the feature value for each observation. Positive SHAP values indicate a higher predicted TG/HDL-C index. B: SHAP dependence plots for Cer16:0, Cer18:0, Cer24:0, and Cer24:1, showing feature value (x-axis) versus SHAP value (y-axis); each point represents one participant. Cer, ceramide; METS‑IR: metabolic score for insulin resistance; SHAP, Shapley Additive Explanations.


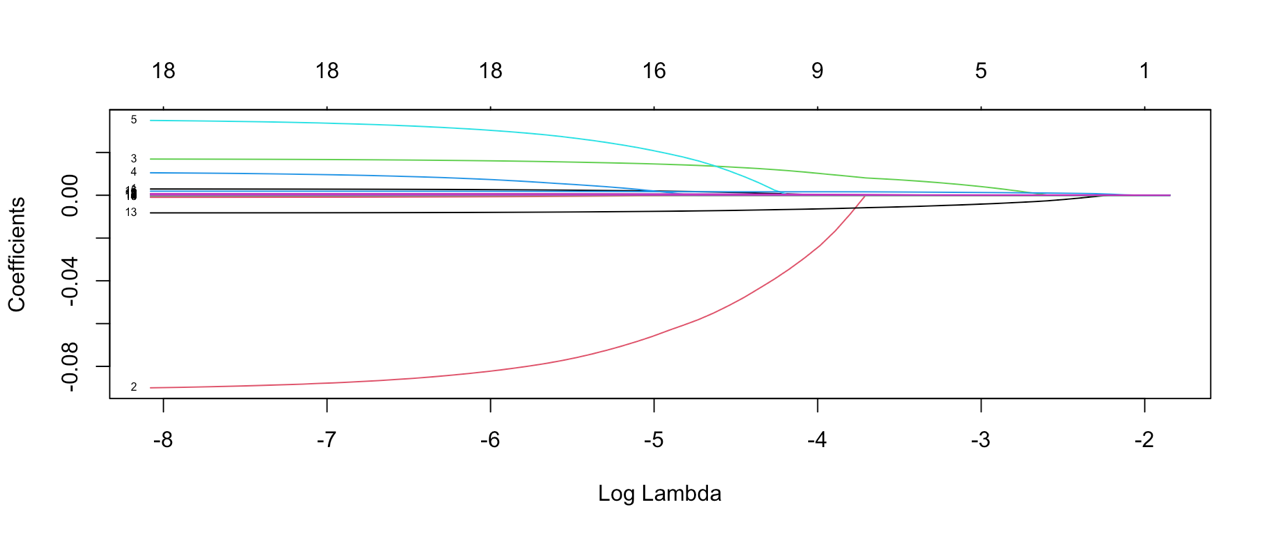
**Supplementary Fig.8 LASSO coefficient paths.** Standardized coefficients are plotted against log(λ); the top axis shows the number of nonzero coefficients.


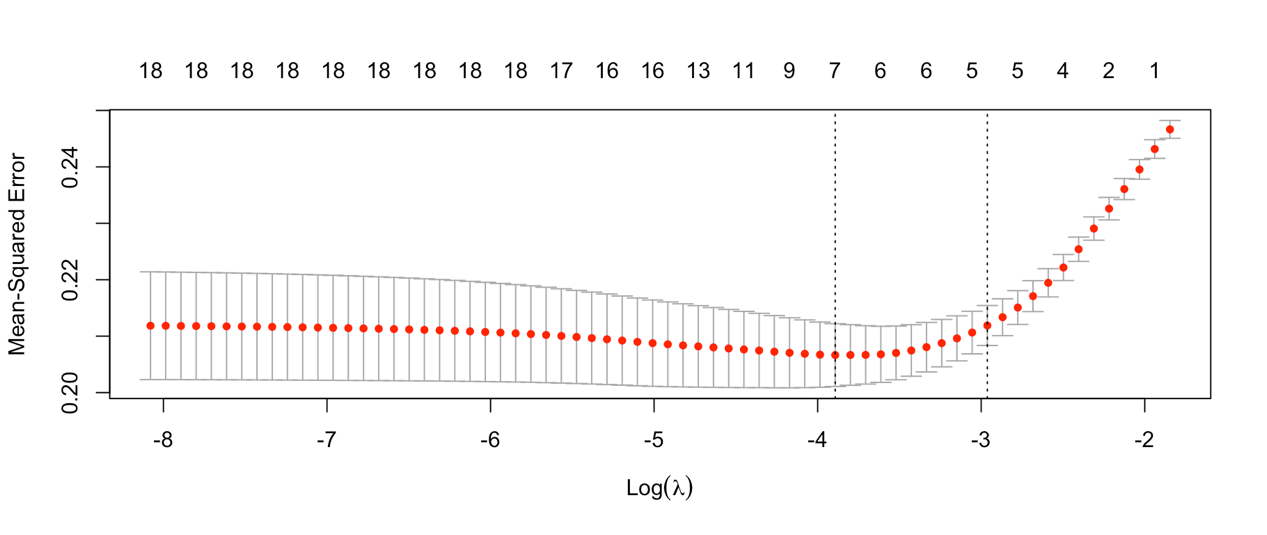


**Supplementary Fig.9 Cross validated mean squared error (MSE) for the least absolute shrinkage and selection operator (LASSO) as a function of log(λ).**


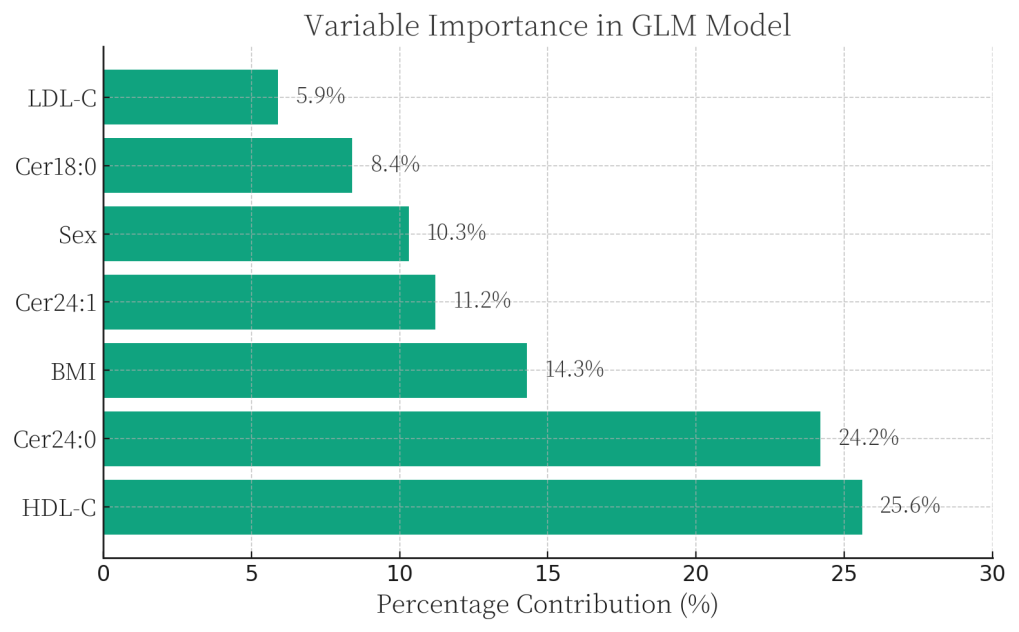


**Supplementary Fig.10 Relative importance of predictors in the final generalized linear model for insulin resistance.** BMI: body mass index; Cer: ceramide; HDL‑C: high‑density lipoprotein cholesterol; LDL‑C: low‑density lipoprotein cholesterol.

| **Supplementary Table 2. Machine learning model performance for ceramide-based IR prediction** | | | |
| --- | --- | --- | --- |
| Model | ROCAUC | Logloss | AUCPR |
| GLM | 0.770 | 0.585 | 0.729 |
| GBM | 0.756 | 0.577 | 0.715 |
| DeepLearning | 0.755 | 0.616 | 0.701 |
| XGBoost | 0.753 | 0.585 | 0.704 |
| DRF | 0.749 | 0.586 | 0.705 |
| XRT | 0.744 | 0.592 | 0.704 |
| AUCPR, Area Under the Precision-Recall Curve; DRF, Distributed Random Forest; GBM, Gradient Boosting Machine; GLM, Generalized Linear Model; IR, Insulin Resistance; Log-loss, Logarithmic Loss; ROCAUC, Receiver Operating Characteristic Area Under the Curve; XGBoost, eXtreme Gradient Boosting; XRT, eXtremely Randomized Trees. | | | |

| **Supplementary Table 3**. **Performance metrics of the model in 5-fold cross-validation** | | | | | | | |
| --- | --- | --- | --- | --- | --- | --- | --- |
| Metric | Mean | SD | CV_1 | CV_2 | CV_3 | CV_4 | CV_5 |
| Accuracy | 0.686 | 0.049 | 0.717 | 0.646 | 0.645 | 0.665 | 0.756 |
| ROC-AUC | 0.764 | 0.037 | 0.784 | 0.775 | 0.706 | 0.751 | 0.803 |
| Precision | 0.607 | 0.046 | 0.613 | 0.581 | 0.576 | 0.579 | 0.685 |
| Recall | 0.859 | 0.092 | 0.88 | 0.947 | 0.773 | 0.944 | 0.753 |
| F1 Score | 0.708 | 0.024 | 0.723 | 0.72 | 0.66 | 0.718 | 0.717 |
| Specificity | 0.541 | 0.151 | 0.6 | 0.369 | 0.541 | 0.435 | 0.759 |
| CV, cross-validation fold; ROC-AUC, area under the receiver operating characteristic curve; SD, standard deviation; | | | | | | | |
